# Quality by Design-Guided Development of *Cynara cardunculus* var. *scolymus* L. Extract-Loaded Liposomes

**DOI:** 10.3390/molecules31193407

**Published:** 2026-09-24

**Authors:** Cristina Ciobanu, Andreea Cornilă, Cobzac Vitalie, Vinicius de Monte Vidal, Victoria Díaz-Tomé, Adriana Trifan, Ioan Tomuță

**Affiliations:** 1 Drug Technology Department, Center for Drug Development, Nicolae Testemițanu State University of Medicine and Pharmacy, 2004 Chisinau, Moldova; 2 Pharmaceutical Technology and Biopharmacy Department, Iuliu Hațieganu University of Medicine and Pharmacy, 400012 Cluj-Napoca, Romania; ioana.an.cornila@elearn.umfcluj.ro (A.C.); tomutaioan@umfcluj.ro (I.T.); 3 The Laboratory of Tissue Engineering and Cell Cultures, Nicolae Testemitanu State University of Medicine and Pharmacy, 2004 Chisinau, Moldova; vitalie.cobzac@usmf.md; 4 Paraquasil Group, Department of Pharmacology, Pharmacy and Pharmaceutical Technology, Institute of Materials iMATUS, Universidade de Santiago de Compostela (USC), 15706 Santiago de Compostela, Spain; vinicius.demonte@rai.usc.es (V.d.M.V.); victoria.diaz@usc.es (V.D.-T.); 5 Department Pharmaceutical Sciences II, Grigore T. Popa University of Medicine and Pharmacy Iasi, 700115 Iasi, Romania; adriana.trifan@umfiasi.ro

**Keywords:** *Cynara cardunculus* var. *scolymus* L., liposomes, Quality by Design, D-optimal experimental design, encapsulation efficiency, lyophilization, stability

## Abstract

An integrated Quality by Design (QbD) approach was developed for the systematic optimization of *Cynara cardunculus* var. *scolymus* L. extraction and its subsequent incorporation into liposomal formulations. A D-optimal design was initially applied to evaluate the influence of extraction method, plant material-to-solvent ratio, and ethanol concentration on total phenolic and flavonoid contents. The optimization identified ultrasound-assisted extraction using 70% ethanol at a plant material-to-solvent ratio of 1:50 as the optimal extraction condition. Phytochemical profiling of the optimized plant extract by LC–TIMS-HRMS/MS led to the putative annotation of 47 metabolites, predominantly hydroxycinnamic acid derivatives and flavonoids. The second D-optimal design elucidated the combined effects of formulation composition and ultrasonic processing and identified an extract-to-phospholipid ratio of 1:0.84 (*w*/*w*), a phospholipid-to-cholesterol ratio of 1:0.42 (*w*/*w*), and probe sonication for 16.73 min as the conditions providing the most favorable balance among particle size, polydispersity index (PDI), encapsulation efficiency (EE), and drug loading. The formulation obtained under these conditions was subsequently evaluated for *in vitro* release, showing distinct release profiles under the two investigated pH conditions over 24 h, with cumulative phenolic release reaching 68.14 ± 3.68% at pH 7.4 and 53.77 ± 2.99% at pH 5.5. The liposomal formulation obtained under optimized conditions, with a particle size of 224.1 ± 1.9 nm, a PDI of 0.206 ± 0.014, and an EE of 75.95 ± 0.24%, was lyophilized with 5% (*w*/*v*) mannitol, with its key physicochemical attributes preserved upon reconstitution. Long-term stability assessment over 12 months showed that the reconstituted lyophilized liposomes retained a particle size below 300 nm, a PDI below 0.30, and an EE above 73%, while TEM analysis confirmed preservation of vesicular morphology. Preliminary cytocompatibility assessment in adult human dermal fibroblasts further indicated a more favorable cellular response to the liposomal formulation than to the free extract. These findings demonstrate the applicability of a systematic approach integrating extract optimization with liposomal formulation development for *C. cardunculus* var. *scolymus* extract-loaded liposomes.

## 1. Introduction

*Cynara cardunculus* var. *scolymus* L. (*C. cardunculus* var. *scolymus*), commonly known as globe artichoke, is a perennial herbaceous plant of the Asteraceae family, native to the Mediterranean basin, and characterized by long-standing ethnopharmacological use and considerable phytochemical diversity [1]. Both the edible immature inflorescences, valued for their nutritional properties, and the leaves, traditionally used in ethnomedicine, contribute to the growing scientific and industrial interest in artichoke phytoconstituents for pharmaceutical and nutraceutical applications [2,3,4,5]. The biological properties of *C. cardunculus* var. *scolymus* are closely associated with its diverse phytochemical composition, particularly phenolic compounds such as caffeoylquinic acid derivatives, flavonoids, tannins, and anthocyanins, alongside sesquiterpene lactones and phytosterols [6,7]. Among these constituents, caffeoylquinic acids and flavonoid derivatives are considered major contributors to the biological properties of globe artichoke [8].

Accessing this rich phenolic profile, however, requires an appropriate extraction strategy, as both the amount and spectrum of compounds recovered from the plant matrix are influenced by solvent composition, solid-to-liquid ratio, and the extraction technique employed [9,10,11,12]. Conventional galenic techniques, such as maceration and percolation, remain attractive because of their simplicity, although they involve longer extraction times and larger solvent volumes, whereas intensified approaches, including turbo extraction and ultrasound-assisted extraction (UAE), can enhance mass transfer and shorten processing time [13,14]. Their performance is nevertheless closely linked to the applied conditions, while excessive mechanical or ultrasonic energy may compromise thermolabile or oxidation-sensitive constituents [15,16]. The concurrent influence of extraction technique, solvent composition, and solid-to-liquid ratio therefore supports their evaluation within a multivariate experimental framework rather than through isolated variation in individual factors.

Beyond the extraction step, the formulation of multicomponent botanical extracts represents an additional technological challenge because their constituents may differ markedly in polarity, molecular structure, and affinity for aqueous or lipid environments [17]. Liposomal systems offer a particularly versatile formulation approach in this context, as their structural organization enables chemically diverse constituents to associate with the aqueous compartment, phospholipid bilayer, or lipid–aqueous interface [18,19]. The feasibility of incorporating complex botanical extracts into vesicular systems has been demonstrated in several studies. Jovanović et al. reported that the liposomal incorporation of a polyphenol-rich *Thymus serpyllum* L. extract was strongly dependent on lipid composition, with sterol content influencing encapsulation efficiency (EE), particle size, polydispersity index (PDI), and storage stability [20]. Similarly, *Macleaya cordata* extract was successfully incorporated into liposomes using a thin-film dispersion method, achieving high encapsulation efficiency and supporting the applicability of this preparation approach to multicomponent botanical extracts [21]. Panomai et al. further demonstrated formulation-dependent behavior for *Oroxylum indicum* leaf extract incorporated into cholesterol-containing niosomal vesicles prepared by thin-film hydration, where optimization of vesicle composition resulted in high encapsulation of phenolic constituents and favorable physicochemical characteristics [22]. Taken together, these studies demonstrate the feasibility of vesicular delivery of complex botanical extracts while highlighting the strong dependence of formulation performance on lipid composition and processing conditions [23]. This complexity may be further influenced by interactions between polyphenolic constituents and phospholipid membranes, which can modify bilayer organization and, consequently, vesicle properties [24,25,26,27,28,29]. These considerations support the need for systematic formulation optimization rather than assuming that vesicular encapsulation will exert comparable effects across chemically diverse botanical extracts. Moreover, because liposomal dispersions remain susceptible to physical and chemical instability during storage, lyophilization is an important stabilization strategy that converts the aqueous dispersions into dry, reconstitutable systems with improved storage characteristics [30,31].

Based on the available literature, studies addressing the liposomal encapsulation of artichoke-derived extracts remain relatively limited [32,33,34]. To the best of our knowledge, the development of *C. cardunculus* var. *scolymus* extract-loaded liposomes has not yet been addressed through an integrated strategy encompassing optimization of the extraction process, systematic formulation development, and long-term stability assessment of the resulting lyophilized system. Quality by Design (QbD) provides a suitable framework for addressing this complexity by establishing relationships between critical material attributes (CMAs), critical process parameters (CPPs), and critical quality attributes (CQAs), thereby enabling the identification of influential variables and their interactions [35,36,37,38,39,40]. For liposomal systems, formulation composition and processing conditions can directly affect key quality attributes such as particle size, size distribution, EE, drug loading (DL), and physicochemical stability [41,42,43,44,45,46,47]. 

Therefore, a QbD approach was selected for the present study to enable systematic identification of the variables governing the CQAs of both the extraction process and the resulting liposomal system. This approach is particularly relevant to complex plant extracts, where compositional variability and formulation parameters may jointly influence vesicle characteristics and payload incorporation. Accordingly, the study aimed to establish the formulation and processing conditions required for the development of *C. cardunculus* var. *scolymus* extract-loaded liposomes, while addressing the preservation of key physicochemical and morphological attributes and the encapsulated payload following lyophilization, reconstitution, and long-term storage.

## 2. Results and Discussion

### 2.1. Quality by Design-Based Development Strategy and Risk Assessment

A preliminary risk assessment was performed to identify the material attributes and process parameters most likely to influence the predefined CQAs of the *C. cardunculus* var. *scolymus* extract-loaded liposomal formulation. The relationships among extract-related factors, formulation composition, processing conditions, and product quality attributes were summarized using an Ishikawa diagram (Figure 1). Based on this assessment, the phytochemical characteristics of the extract, lipid composition, and ultrasonic processing conditions were selected for further experimental investigation. Plant-related sources of variability, including botanical origin, harvest period, drying conditions, and powdering, were controlled by using plant material from the same source and processed under standardized conditions [48,49,50].

Extraction and liposomal formulation variables were subsequently investigated experimentally. Following formulation optimization, lyophilization was applied as a post-processing stabilization step using a fixed protocol. Because lyophilization parameters were not included as variables in the QbD, their effects were assessed separately through post-lyophilization characterization and long-term stability evaluation. 

### 2.2. Optimization of the Extraction Process

Optimization of the extraction process was performed as the first experimental stage to obtain a phytochemically characterized extract suitable for subsequent liposomal formulation. The extraction method (X1), plant material-to-solvent ratio (X2), and ethanol concentration in the hydroethanolic extraction medium (X3) were investigated as independent variables, while total phenolic content (TPC; Y1) and total flavonoid content (TFC; Y2) were selected as response variables. The experimental responses were fitted using partial least squares (PLS) regression. Model performance was evaluated using R^2^, adjusted R^2^, Q^2^, model validity, and reproducibility. The TPC model showed R^2^ = 0.945, adjusted R^2^ = 0.915, Q^2^ = 0.796, model validity = 0.602, and reproducibility = 0.978. The corresponding values for the TFC model were R^2^ = 0.944, adjusted R^2^ = 0.903, Q^2^ = 0.589, model validity = 0.493, and reproducibility = 0.984 (Figure 2). 

The close agreement between R^2^ and adjusted R^2^, together with the positive Q^2^ values and high reproducibility, supported the adequacy of the fitted models for describing the experimental responses within the investigated design space. The standardized model coefficients and their confidence intervals are presented in Figures S1 and S2 for TPC and TFC, respectively. The coefficient plots revealed distinct contributions of the investigated extraction variables to both responses. The plant material-to-solvent ratio (X2) showed a negative coefficient for TPC and TFC, while ethanol concentration (X3) exerted a positive effect, particularly on TFC. Interaction effects between the investigated extraction variables were also observed. The combined effects of X2 and X3 under UAE conditions were further visualized using three-dimensional response surface plots (Figure 3).

Across the 21 experimental runs, TPC ranged from 0.136 to 0.635 mg/mL, while TFC ranged from 0.046 to 0.261 mg/mL, demonstrating substantial variation among the investigated extraction conditions. Based on the combined evaluation of the experimental responses and fitted models, UAE was selected as the most suitable extraction method among those investigated. Multi-response optimization identified UAE using 70% ethanol and a plant material-to-solvent ratio of 1:50 as the optimal extraction conditions, providing the most favorable combined TPC and TFC responses within the investigated experimental domain. The extract obtained under these optimized conditions was subsequently selected for phytochemical characterization and incorporation into the liposomal formulation.

### 2.3. Phytochemical Profile of the Cynara cardunculus var. scolymus L. Optimized Extract Revealed by LC–TIMS-HRMS/MS

Following optimization of the extraction process, the extract obtained under the selected optimal conditions (UAE, 70% ethanol, and a plant material-to-solvent ratio of 1:50, corresponding to 1 g of plant material per 50 mL of solvent) was concentrated to dryness by rotary evaporation and subsequently characterized by LC–TIMS-HRMS/MS to establish its phytochemical profile. Untargeted liquid chromatography–trapped ion mobility spectrometry–high-resolution tandem mass spectrometry (LC–TIMS-HRMS/MS) analysis provided a comprehensive characterization of the phytochemical composition of the *C. cardunculus* var. *scolymus* leaf extract. A total of 47 metabolites were putatively annotated based on accurate mass measurements, isotopic distribution, collision cross-section (CCS) values and MS/MS fragmentation patterns using the MetaboScape 2024b platform in combination with spectral databases and literature data [51]. Representative metabolites from the major phytochemical classes annotated in the optimized extract are presented in Table 1.

The annotated metabolites belonged predominantly to hydroxycinnamic acid derivatives and flavonoids, the major phenolic classes reported in *Cynara* species [52,53]. Hydroxycinnamic acid derivatives included quinic acid, chlorogenic acid, dicaffeoylquinic acid, caffeic acid, p-coumaric acid, and isoferulic acid. This profile is consistent with our previous LC-HRMS/MS characterization of *Cynara* varieties, in which mono- and dicaffeoylquinic acids represented a predominant class of specialized metabolites [6]. Chlorogenic acid, a characteristic hydroxycinnamic acid of *C. cardunculus* var. *scolymus*, has been associated with the antioxidant, hepatoprotective, and hypolipidemic properties of artichoke preparations [54,55]. Flavonoids were mainly represented by glycosylated derivatives of quercetin and kaempferol, including quercetin-O-dihexoside, quercetin-O-hexoside, kaempferol-O-rutinoside (nicotiflorin), kaempferol-O-hexoside, quercetin-O-deoxyhexoside, and kaempferol-O-deoxyhexoside, together with the corresponding aglycones. These flavonoid derivatives are widely distributed in higher plants and have been associated with antioxidant, anti-inflammatory, and cytoprotective activities [56,57].

The untargeted LC–TIMS-HRMS/MS analysis confirmed a phenolic profile dominated by hydroxycinnamic acid derivatives and flavonoid glycosides, consistent with the established phytochemical composition of *C. cardunculus* var. *scolymus*.

### 2.4. Optimization of Liposomal Formulation by Design of Experiments

Following selection and phytochemical characterization of the optimized extract, a second Design of Experiments (DoE) was applied to investigate the influence of formulation composition and ultrasonic processing conditions on the characteristics of the liposomal system. The investigated factor ranges and processing conditions were selected based on preliminary formulation trials, which were used to define an experimentally feasible domain for the subsequent DoE study. The investigated factors included the extract-to-phospholipid ratio (X1), phospholipid-to-cholesterol ratio (X2), ultrasonic processing method (X3; A, probe sonication only; B, combined probe sonication and ultrasonic bath treatment; C, ultrasonic bath treatment only), and total ultrasonic processing time (X4). Particle size, PDI, DL, and EE were evaluated as response variables before and after sonication. To ensure reliable assessment of the payload-related responses, all 15 experimental formulations were purified by dialysis prior to characterization, in accordance with the recognized need to separate non-encapsulated constituents from the liposomal fraction before payload quantification [58,59]. The formulations showed substantial variability across the investigated responses. Before sonication, particle size ranged from 415.8 to 1596 nm and PDI from 0.404 to 1.025 while EE and DL ranged from 35.25% to 85.58% and 16.71% to 37.18%, respectively. After sonication, particle size ranged from 129.2 to 975.0 nm and PDI from 0.198 to 0.753, whereas EE and DL ranged from 25.22% to 82.78% and 14.32% to 34.66%, respectively. The observed variability provided the experimental basis for evaluating the factor–response relationships and subsequent multi-response optimization. 

The summary-of-fit statistics (Figure 4) further supported the adequacy of the developed PLS models. The R^2^ and reproducibility values, together with the Q^2^ estimates, indicated an appropriate description of the experimental data and supported the use of the models for interpretation and optimization within the investigated experimental domain. The standardized regression coefficients (Figures S3–S6) provided further insight into the magnitude and direction of the estimated effects and their interactions, revealing response-specific patterns associated with formulation composition and ultrasonic processing conditions.

The response surface plots were used to further visualize the combined effects of the two formulation variables, namely the plant extract-to-phospholipid ratio (X1) and the phospholipid-to-cholesterol ratio (X2), on the investigated liposomal characteristics (Figure 5). For graphical representation of these relationships, the ultrasonic processing time (X4) was fixed at 15 min. The response surfaces illustrate the predicted changes in particle size, PDI, EE, and DL across the investigated formulation domain, both before and after sonication. Within this experimental region, increasing the extract-to-phospholipid ratio was associated with higher EE and DL, whereas increasing the phospholipid-to-cholesterol ratio tended to reduce these responses. The corresponding surfaces for particle size and PDI further showed that the physicochemical characteristics of the vesicles varied across the investigated formulation domain.

Multi-response optimization using the desirability function identified an optimal extract-to-phospholipid composition of 54.33:45.67 (*w*/*w*), corresponding to a ratio of 1:0.84, and a phospholipid-to-cholesterol composition of 70.5:29.5 (*w*/*w*), corresponding to a ratio of 1:0.42, probe sonication only (Method A), and a total ultrasonic processing time of 16.73 min. These conditions represented the optimal compromise between minimizing particle size and PDI and maximizing EE and DL and were therefore selected for subsequent lyophilization and stability studies. These effects may be attributed, at least in part, to the influence of cholesterol on lipid bilayer organization, fluidity, and permeability [60], together with the contribution of phospholipid composition to vesicle formation and size [61]. In addition, phytochemical constituents of the incorporated extract may interact with phospholipid membranes through non-covalent interactions, including hydrogen bonding and hydrophobic interactions, thereby affecting membrane organization and physicochemical properties [62].

### 2.5. In Vitro Release Profile of the Optimized Liposomal Formulation

The *in vitro* release profiles of phenolic constituents from the optimized liposomal formulation differed between the near-neutral and mildly acidic conditions investigated (Figure 6), indicating an influence of medium pH on the release behavior of the encapsulated payload.

During the initial 3 h, cumulative release increased progressively under both conditions, reaching 40.02 ± 2.32% at pH 7.4 and 37.47 ± 1.76% at pH 5.5. A more pronounced difference between the release profiles became apparent thereafter. At pH 7.4, cumulative release reached 63.61 ± 3.13% after 5 h and subsequently approached a plateau, reaching 68.14 ± 3.68% after 24 h. At pH 5.5, a more gradual release profile was observed, with cumulative release reaching 47.68 ± 1.02% at 5 h and 53.77 ± 2.99% after 24 h. The differences observed between the two profiles further indicate that the release environment influenced the release of the encapsulated phenolic payload. This finding is consistent with previous reports emphasizing the importance of release-medium composition and experimental conditions in the assessment of release from liposomal systems [63,64]. Differences in cumulative release under acidic and near-neutral conditions have been reported for polyphenol-loaded carriers, although the magnitude and direction of this effect appear to depend on the physicochemical characteristics of the encapsulated compounds and the composition and structural properties of the carrier [65,66]. These observations support the interpretation that the distinct release profiles observed herein at pH 5.5 and 7.4 reflect the interplay between the release medium, the complex phenolic payload, and the liposomal matrix rather than a simple pH-responsive behavior. 

### 2.6. Lyophilization and Stability Studies 

Lyophilization is widely employed to improve the long-term stability of liposomal formulations by reducing the physical instability associated with aqueous dispersions, including aggregation, fusion, and precipitation [67,68,69,70,71,72]. Following DoE optimization, three independent batches of the selected liposomal formulation were prepared under the optimized formulation and processing conditions. Prior to lyophilization, the optimized liposomal dispersions were purified by dialysis and characterized to establish their initial physicochemical attributes. The purified formulations showed a mean particle size of 224.1 ± 1.9 nm, a PDI of 0.206 ± 0.014, a ZP of −2.205 ± 0.01 mV, and an EE of 75.95 ± 0.24% (Table 2). These values were used as the pre-lyophilization reference for assessing changes associated with freeze-drying and subsequent reconstitution. Following lyophilization with 5% (*w*/*v*) mannitol and reconstitution, the physicochemical characteristics of the formulation remained comparable to the pre-lyophilization values, with a particle size of 224.3 ± 4.0 nm, PDI of 0.203 ± 0.021, ZP of −2.206 ± 0.16 mV, and EE of 75.92 ± 1.09% (Table 2). The limited changes observed immediately after lyophilization and reconstitution support the suitability of the selected freeze-drying conditions and the use of 5% (*w*/*v*) mannitol as cryoprotectant, consistent with previous reports describing its protective role [73,74].

During the 12-month stability study, storage time significantly affected particle size (*p* < 0.001), with the value at 12 months being significantly higher than those at 0, 3, and 6 months after Holm adjustment (*p* < 0.05). A significant effect was also observed for PDI (*p* = 0.027), although pairwise differences were not significant after multiple-comparison adjustment. ZP showed a significant change (*p* = 0.015), with a significant difference between 0 and 12 months (adjusted *p* = 0.045), whereas EE and residual moisture content remained statistically unchanged (*p* = 0.145 and *p* = 0.094, respectively). EE remained above 73% throughout the study.

Despite the near-neutral zeta potential values (approximately −2 mV), the formulation retained acceptable colloidal characteristics after reconstitution. In liposomes composed of hydrogenated phosphatidylcholine and cholesterol loaded with a phenolic extract, such behavior may reflect contributions from steric and interfacial effects in addition to electrostatic interactions. Cholesterol increases bilayer rigidity and packing density, while the phenolic constituents of the extract may interact with the polar headgroup region and the upper portion of the acyl chains. Such interactions may limit the close approach of vesicles and reduce the tendency toward irreversible aggregation, even when surface charge is low [75,76]. The limited increase in hydrodynamic diameter observed only at the 12-month time point, together with the preservation of particle size below 300 nm, PDI below 0.30, and encapsulation efficiency above 73% throughout storage, supports this interpretation. The low residual moisture content maintained throughout the 12-month storage period further supports the quality of the lyophilized formulation [77]. 

TEM examination further supported the morphological preservation of the liposomal vesicles after lyophilization and reconstitution during storage (Figure 7). At 3, 6, and 12 months, the samples showed predominantly spherical vesicular structures with clearly distinguishable boundaries. Although local vesicle clustering was visible in the micrographs, no widespread structural collapse or marked loss of vesicular morphology was evident over the investigated storage period. These observations complement the physicochemical stability data, indicating preservation of the vesicular architecture after reconstitution, despite the increase in hydrodynamic particle size detected by dynamic light scattering measurements (DLS) at 12 months.

### 2.7. In Vitro Cytocompatibility Assessment on Human Dermal Fibroblasts

Following physicochemical optimization of the liposomal formulation, a preliminary cytocompatibility assessment was performed to determine whether incorporation of *C. cardunculus* var. *scolymus* extract into the optimized liposomal system influenced the cellular response compared with the corresponding free extract. Adult human dermal fibroblasts were exposed to the free extract and the optimized extract-loaded liposomal formulation at concentrations ranging from 5 to 400 µg/mL for 24, 48, and 72 h, and cellular metabolic activity was evaluated using the 3-(4,5-dimethylthiazol-2-yl)-2,5-diphenyltetrazolium bromide (MTT) assay. Exposure to the free extract resulted in a concentration- and time-dependent reduction in relative cell viability (Figure 8). After 24 h, viability ranged from 47.05% at 400 µg/mL to 100.07% at 5 µg/mL. At concentrations ≤50 µg/mL, relative cell viability ranged from 89.61% to 100.07%, whereas concentrations of 100–400 µg/mL resulted in a more pronounced reduction (47.05–68.70%). Prolonged exposure further decreased cellular metabolic activity, with viability ranging from 26.02% to 55.65% after 48 h and from 14.35% to 47.15% after 72 h. The concentration- and time-dependent response observed for the free extract may reflect the complex cellular effects of its phytochemical constituents. Among these, chlorogenic acid, identified in the present study, has previously been investigated for its effects in fibroblast models [78].

The extract-loaded liposomal formulation showed a more favorable cytocompatibility profile. Relative cell viability ranged from 81.05% to 112.42% after 24 h, from 62.61% to 108.77% after 48 h, and from 48.21% to 91.12% after 72 h. At 24 h, relative cell viability was significantly higher for the extract-loaded liposomal formulation than for the free extract at 400 and 200 µg/mL (Holm-adjusted *p* < 0.05), whereas no significant differences were observed at the other tested concentrations. At 48 and 72 h, significantly higher relative cell viability was observed for the liposomal formulation at all tested concentrations from 5 to 400 µg/mL (Holm-adjusted *p* < 0.05). These findings indicate that incorporation of the extract into the liposomal formulation was associated with a more favorable cytocompatibility profile than that of the free extract, particularly after prolonged exposure. Notably, previous studies have evaluated the cytocompatibility of hydroalcoholic artichoke-derived extracts in fibroblast models, including NIH/3T3 mouse fibroblasts exposed to extracts obtained from globe artichoke agro-wastes [79] and HFF-1 human fibroblasts treated with a hydroalcoholic leaf extract of *C. cardunculus* subsp. *cardunculus* (wild artichoke) [80]. The present study extends these observations by evaluating the cytocompatibility of a liposomal formulation containing *C. cardunculus* var. *scolymus* extract in human dermal fibroblasts, thereby providing evidence on the cellular compatibility of the encapsulated extract in a human skin-derived cell model. Nevertheless, these findings should be regarded as preliminary evidence that liposomal incorporation may improve the cytocompatibility of the extract, rather than as confirmation of biological efficacy.

## 3. Materials and Methods

### 3.1. Materials

#### 3.1.1. Plant Material

Leaf material of *Cynara cardunculus* var. *scolymus* L. was obtained from plants grown from certified commercial seeds purchased from Jelitto Perennial Seeds (Germany; lot no. CA70570029034AA2AA0s) and cultivated in the medicinal plant collection of “Nicolae Testemițanu” State University of Medicine and Pharmacy, Chișinău, Republic of Moldova. Botanical identification was performed based on macroscopic and microscopic characteristics. A voucher specimen was deposited in the Herbarium of the Faculty of Pharmacy, “Nicolae Testemițanu” State University of Medicine and Pharmacy, Chișinău, Republic of Moldova, under voucher code CC.2025.2.1.25.

#### 3.1.2. Chemicals and Reagents

The gallic acid (≥98.0%) and rutin (≥94%) standards and Folin–Ciocâlteu reagent used for quantitative determination by UV-Vis spectrophotometry were purchased from Sigma-Aldrich (Schnelldorf, Germany). Sodium acetate and sodium bicarbonate were purchased from Honeywell Fluka (Seelze, Germany), while anhydrous aluminum chloride (99% purity) was purchased from Thermo Fisher Scientific (Waltham, MA, USA). For the preparation of liposomes, cholesterol from ovine wool (≥92.5% purity, Sigma-Aldrich, St. Louis, MO, USA) and hydrogenated soybean phosphatidylcholine (Phospholipon^®^ 90 H, Lipoid GmbH, Ludwigshafen, Germany) with a phospholipid content ≥90.0% (CAS No. 97281-48-6) were used. All solvents and chemical reagents used were of analytical-grade or higher. 

### 3.2. Design of Experiments and Quality by Design Strategy

The experimental development followed a sequential optimization strategy comprising two DoE stages. The first DoE was applied to optimize the extraction of phenolic constituents from *C. cardunculus* var. *scolymus* leaves by evaluating the effects of extraction method, plant material-to-solvent ratio, and ethanol concentration on TPC and TFC. The extract obtained under the selected optimal conditions was subsequently characterized by LC–TIMS-HRMS/MS and used for liposomal formulation development.

Following selection and phytochemical characterization of the optimized extract, a QbD framework was applied to guide the development of the extract-loaded liposomal formulation in accordance with the principles outlined in ICH Q8(R2) and ICH Q9 [81,82]. The QTPP was established based on the intended characteristics of the liposomal formulation, and particle size, PDI, EE, and DL were identified as CQAs. For the optimization process, a particle size below 300 nm and a PDI below 0.30 were defined as target criteria, while EE and DL were targeted for maximization [83,84].

ZP was monitored as an additional physicochemical attribute but was not selected as a response variable in the second DoE, as preliminary measurements of the investigated phospholipid–cholesterol systems consistently yielded low-magnitude, near-neutral values and therefore provided limited discriminatory value for optimization. This behavior was considered compatible with the predominantly non-ionic nature of the lipid components used in the formulation. ZP was nevertheless monitored for the optimized formulation and throughout the stability study to detect potential changes in vesicle surface characteristics.

### 3.3. Development of C. cardunculus var. scolymus Extract

A D-optimal fractional experimental design was employed to optimize the extraction of phenolic compounds from *C. cardunculus* var. *scolymus* leaves. Three independent variables were investigated: extraction method (X1; classical maceration, turbo-extraction, and UAE), plant material-to-solvent ratio (X2; 1:50, 1:100, or 1:200 g/mL), and ethanol concentration in the hydroethanolic solvent (X3; 30%, 50%, or 70%). The experimental design comprised 21 runs (Table 3). The investigated solid-to-solvent ratios were selected to ensure adequate wetting of the dried, fibrous and bulky leaf material while allowing assessment of the effect of increasing solvent availability on extraction performance. The ethanol concentration range (30–70%, *v*/*v*) was selected to cover hydroethanolic conditions commonly used for the recovery of phenolic constituents while allowing assessment of the effect of solvent polarity on extraction performance.

Each extraction method was performed under its specific operating conditions. Classical maceration was carried out at room temperature for 10 days in a closed vessel, with manual agitation three to four times daily, according to the general procedure described in the Romanian Pharmacopoeia [85]. For turbo-extraction, the plant material was pre-soaked in the corresponding hydroethanolic solvent for 1 h and then processed using a Turbotest^®^ Up turbo-extractor (VMI, Montaigu-Vendée, France) at 4000 rpm for 10 min at 25 ± 2 °C. UAE was performed using a UP100H ultrasonic processor (Hielscher Ultrasonics GmbH, Teltow, Germany) at 100% amplitude for 30 min under a pulsed regime with 1-min intervals, based on conditions established in our previous study [86], while the extraction temperature was maintained at 40 °C using a thermostatically controlled water bath (IncuMax).

The optimized liquid extract obtained under the selected extraction conditions was subsequently concentrated to dryness using a rotary evaporator (Heidolph, Germany) at 45 °C and 100 rpm under reduced pressure until complete solvent removal. The resulting dry extract was used for subsequent liposomal formulation studies.

### 3.4. Untargeted LC–TIMS-HRMS/MS Metabolite Profiling

Untargeted metabolite profiling was performed using a high-resolution liquid chromatography–tandem mass spectrometry (LC–TIMS-HRMS/MS) platform consisting of an ELUTE UPLC system (Bruker Daltonics, Bremen, Germany) coupled to a timsTOF Pro mass spectrometer equipped with a VIP-HESI electrospray ion source and a tims-QTOF mass analyzer. Analyses were carried out at the University of Santiago de Compostela (USC, Santiago de Compostela, Spain). The extract was dissolved in methanol to obtain a final concentration of 1000 µg/mL, filtered through a 0.22 µm polytetrafluoroethylene membrane, and injected (5 µL) into the chromatographic system. Chromatographic separation was performed on an Acquity UPLC BEH C18 column (2.1 × 50 mm, 1.7 µm; Waters, Milford, MA, USA) maintained at 40 °C. The mobile phase consisted of water containing 0.1% formic acid (phase A) and acetonitrile with 0.1% formic acid (phase B). An isocratic elution at 80:20 (A:B) was maintained at a flow rate of 0.5 mL/min. Mass spectrometric detection was carried out using electrospray ionization operating in negative-ion mode. MS and MS/MS spectra were acquired using Parallel Accumulation Serial Fragmentation (PASEF) over an m/z range of 20 to 1300. Data processing and interpretation were performed using MetaboScape 2024b software (version 13.0.3; Bruker Daltonics GmbH & Co., Bremen, Germany) for feature detection, alignment and metabolite annotation. Compound annotations were based on accurate mass, isotopic pattern, collision cross-section (CCS) values and MS/MS fragmentation data, using the integrated MetaboScape workflow combined with spectral databases and published literature.

### 3.5. Total Phenolic Content Determination

The total phenolic content (TPC) was determined using the Folin–Ciocalteu (FC) method described by Singleton and Rossi, with some modifications [87]. An aliquot of 300 µL of extract was mixed with 150 µL of FC reagent (diluted 1:10). After incubation for 10 min at room temperature, 1.2 mL of 10% sodium bicarbonate solution and 1.35 mL of purified water were added. The samples were subsequently incubated in the dark for 45 min. Absorbance was measured at 765 nm using a Specord^®^ 200 Plus spectrophotometer (Analytik Jena GmbH+Co. KG, Jena, Germany) against a blank solution. A calibration curve was constructed using gallic acid at concentrations ranging from 0 to 115 µg/mL. The results were expressed as mg/mL of gallic acid equivalents (GAEs).

### 3.6. Determination of Total Flavonoid Content

The total flavonoid content was determined spectrophotometrically according to the aluminium chloride colorimetric method [85,88]. Samples were dissolved in methanol and mixed with 0.1 mL of 10% aluminium chloride hexahydrate, 0.1 mL of 1 M sodium acetate and 2.8 mL of deionized water. After 20 min of incubation at room temperature, the absorbance of the resulting solution was determined spectrophotometrically at 435 nm. Rutin was chosen as a standard (concentration range: 0.05–0.1 mg/mL) and the total flavonoid content was expressed in mg/mL of rutin equivalents (REs).

### 3.7. Development of liposomes loaded with Cynara cardunculus var. scolymus L. Extract

#### 3.7.1. Experimental Design

A multifactorial DoE approach was employed to investigate the effects of formulation and processing variables on the CQAs of the extract-loaded liposomes. Prior to the D-optimal design, preliminary formulation trials were conducted by varying the solvent system, lipid composition, and sonication conditions, with formulation feasibility assessed based on physical appearance and particle size. Based on these preliminary trials, four independent variables were selected for optimization: extract-to-phospholipid composition (X1), phospholipid-to-cholesterol ratio (X2), ultrasonic processing method (X3), and total ultrasonic processing time (X4). X1 was investigated over an extract-to-phospholipid mass composition range of 35:65 to 65:35 (*w*/*w*). The phospholipid-to-cholesterol ratio (X2) was investigated at three levels (1:2, 1:1, and 2:1), expressed as parts by weight of phospholipid to cholesterol. The numerical values used in the D-optimal model represent the software parameterization of these experimental levels. X3 was included as a categorical factor comprising probe sonication (A), combined probe sonication with ultrasonic bath treatment (B), and ultrasonic bath treatment (C). X4 was evaluated as a continuous factor over the range of 9–21 min. The selected factor levels were chosen to cover a technologically relevant formulation and processing domain, enabling systematic evaluation of the effects of lipid composition, extract loading, sonication method, and processing time on the investigated CQAs. The D-optimal design comprised 15 experimental formulations (Table 4), including three center-point replicates to estimate experimental variability and assess reproducibility. Formulations were prepared in randomized order to minimize systematic experimental bias.

The selected responses comprised particle size, PDI, DL, and EE, determined before and after sonication. The experimental design enabled assessment of the individual and combined effects of formulation composition and ultrasonic processing conditions on the investigated liposomal CQAs. Optimization criteria were defined to minimize particle size and PDI while maximizing DL and EE.

#### 3.7.2. Liposome Preparation

Liposomes loaded with *C. cardunculus* var. *scolymus* extract were prepared using the thin-film hydration method. Briefly, phospholipids and cholesterol were accurately weighed according to the experimental design matrix and dissolved in ethanol. The solvent was removed under reduced pressure using a rotary evaporator (Heidolph, Germany) at 100 rpm at 45 °C, leading to the formation of a thin lipid film on the inner wall of the evaporation flask. The lipid film was further maintained under reduced pressure to ensure complete removal of residual solvent.

The dried lipid film was subsequently hydrated with the aqueous phase containing plant extract prepared using ultrapure water, previously characterized according to its total phenolic content and total flavonoid content. Hydration was performed without pH adjustment, under controlled conditions at 100 rpm and 40 °C, to facilitate lipid swelling and vesicle formation [89]. The resulting liposomal dispersions were then subjected to ultrasonic size-reduction treatment according to the experimental design (Table 4). The total ultrasonic processing time varied between 9 and 21 min, according to the design matrix. 

Following preparation of the 15 experimental liposomal formulations according to the D-optimal design, each dispersion was purified by dialysis to remove non-encapsulated extract constituents. Purification was performed using pre-wetted, ready-to-use SnakeSkin™ Dialysis Tubing (MWCO 10 kDa, internal diameter 22 mm; Thermo Fisher Scientific, Waltham, MA, USA). The entire volume of each liposomal formulation was transferred into the dialysis tubing and dialyzed against 500 mL of ultrapure water under continuous magnetic stirring at 150 rpm and room temperature (22 ± 2 °C) for 24 h. The external medium was completely replaced with 500 mL of fresh ultrapure water after 1, 4, and 8 h to maintain the concentration gradient across the dialysis membrane and facilitate the removal of non-encapsulated extract constituents. The dialysis system was protected from light with aluminum foil throughout the entire dialysis period. Following dialysis, the purified dispersions were collected and immediately subjected to characterization.

#### 3.7.3. Particle Size, Polydispersity Index and Zeta Potential

Particle size, PDI, and ZP of the liposomes were characterized using a Zetasizer Nano ZS90 (Malvern Panalytical Ltd., Malvern, UK) at 25 °C. A sample from each formulation was diluted with ultrapure water in a 1:10 ratio to avoid measurement errors. Each sample was measured in triplicate, and the results were expressed as mean ± standard deviation (SD).

#### 3.7.4. Determination of Encapsulation Efficiency

Following dialysis purification, an aliquot of 0.1 mL of the purified liposomal dispersion was diluted with 0.9 mL of distilled water and centrifuged at 15,000 rpm for 10 min at 4 °C using a 3-30 KS refrigerated centrifuge (Sigma Laborzentrifugen GmbH, Osterode am Harz, Germany). The supernatant was collected, and a 10 µL aliquot was further diluted with 990 µL of 96% ethanol to disrupt residual liposomal structures and release the associated phenolic compounds. The resulting solution was centrifuged again at 15,000 rpm for 10 min to remove any remaining particulate matter prior to spectrophotometric analysis. TPC was determined using the FC method described in Section 3.5. The EE was calculated according to Equation (1):

EE (%) = [W_enc_/W_total_] × 100 (1)

where W_enc_ represents the TPC recovered from the purified liposomal fraction after liposome disruption, and W_total_ represents the initial amount of phenolic compounds introduced into the formulation. All measurements were performed in triplicate, and results are expressed as mean ± standard deviation (SD).

#### 3.7.5. Determination of Drug Loading 

The DL was calculated based on the amount of encapsulated phenolic constituents quantified in the purified liposomal formulation. Briefly, following dialysis purification, 0.5 mL of the liposomal dispersion was centrifuged at 15,000 × g for 30 min at 4 °C to recover the liposomal fraction. The resulting liposomal pellet was disrupted by adding 4.5 mL of absolute methanol, followed by sonication for 10 min in an ultrasonic bath and vortexing for 5 min to ensure complete disruption of the liposomes and release of the encapsulated phenolic compounds. TPC in the lysed liposomal fraction was quantified using the FC method, as described in Section 3.5. Absorbance was measured at 765 nm using a Specord^®^ 200 Plus UV–Vis spectrophotometer (Analytik Jena GmbH+Co. KG, Jena, Germany), and the results were expressed as gallic acid equivalents (GAEs). Drug loading was calculated according to Equation (2):

DL (%) = [Mass of encapsulated extract/(Total mass of lipids + encapsulated extract)] × 100(2)

where the mass of encapsulated extract represents the amount of phenolic compounds quantified in the purified liposomal fraction after liposome disruption. The total lipid mass represents the combined mass of phospholipid and cholesterol used in the formulation. All measurements were performed in triplicate, and the results are expressed as mean ± standard deviation (SD).

### 3.8. In Vitro Release Study

The *in vitro* release study was performed using the dialysis membrane method in three independent experiments for each of the two pH conditions (n = 3), with each sample analyzed in triplicate. A 14 kDa MWCO membrane was selected based on our previous studies for the *in vitro* release assessment of plant extract liposomal formulations, consistent with literature reports [90]. Prior to the release experiments, cellulose dialysis bags (MWCO 14 kDa, average flat width 25 mm; Sigma-Aldrich, St. Louis, MO, USA) were prepared according to the manufacturer’s instructions. Briefly, 2 mL of the optimized extract-loaded liposomal formulation was transferred into each dialysis bag. For each pH condition, the dialysis bags were individually immersed in 100 mL of the corresponding release medium, consisting of phosphate-buffered saline (PBS) at pH 7.4 or sodium acetate buffer at pH 5.5. The release experiments were conducted at 37 °C under continuous magnetic stirring at 75 rpm. At predetermined time points (1, 2, 3, 4, 5, 6, 7, 8, and 24 h), 1 mL aliquots were withdrawn from the release medium and immediately replaced with an equal volume of fresh medium maintained under the same experimental conditions [63]. The collected samples were treated with absolute ethanol and centrifuged at 15,000 rpm for 15 min at 4 °C using a 3-30 KS refrigerated centrifuge (Sigma Laborzentrifugen GmbH, Osterode am Harz, Germany). The supernatants were collected, and the released TPC was determined spectrophotometrically using a Specord^®^ 200 Plus spectrophotometer (Analytik Jena GmbH+Co. KG, Jena, Germany), as described in Section 3.5.

The cumulative amount of phenolic compounds released at each sampling time was corrected for the sequential withdrawal and replacement of the release medium according to Equation (3):

(3) $$Q_{n}=V\;C_{n}+v\sum_{i=1}^{n-1}C_{i}$$

where Q_n_ is the cumulative amount released at sampling time n, V is the total volume of the release medium (100 mL), C_n_ is the TPC concentration in the release medium at time n, v is the volume withdrawn at each sampling point (1 mL), and C_i_ represents the TPC concentration in each previously withdrawn sample. 

Cumulative release was expressed as a percentage of the initial TPC amount present in the liposomal dispersion introduced into the dialysis membrane (*M*_0_), according to Equation (4):

(4) $$\mathrm{Cumulative}\;\mathrm{release}\;\left(\%\right)=\;\left(\frac{\mathrm{Qn}}{M_{0}}\right)\times \;100$$

where *M*_0_ represents the initial TPC amount (108.72 µg GAE) contained in the 2 mL of purified liposomal formulation used for the release experiment, corresponding to an initial TPC concentration of 54.36 µg GAE/mL. 

### 3.9. Lyophilization of the Optimized Liposomal Formulation

Preliminary lyophilization trials were performed using mannitol at concentrations of 2.5%, 5%, and 7.5% (*w*/*v*) as a cryoprotectant. Following freeze-drying and reconstitution, the formulations were evaluated by DLS in terms of particle size, PDI, and ZP. Among the tested concentrations, 5% (*w*/*v*) mannitol provided the most favorable characteristics, yielding a mean particle size of 224.15 nm and a PDI of 0.20, and was therefore selected for the subsequent lyophilization experiments.

Three batches of liposomes containing *C. cardunculus* var. *scolymus* extract were subsequently prepared according to the optimized formulation. The liposomal dispersions were purified by dialysis using SnakeSkin™ Dialysis Tubing (MWCO 10 kDa, average flat width 22 mm) against ultrapure water under magnetic stirring at 150 rpm at room temperature for 24 h.

Following purification, aliquots of 1 mL from each liposomal batch were transferred into vials and freeze-dried using 5% (*w*/*v*) mannitol as a cryoprotectant. Lyophilization was performed using a bench-top freeze-dryer (Advantage Plus, SP Scientific, Warminster, PA, USA) according to the following protocol: freezing at −55 °C for 18 h, primary drying at −25 °C and 200 mTorr for 24 h, followed by secondary drying at 20 °C and 400 mTorr for 12 h.

### 3.10. Stability Studies 

Three independent batches of the optimized formulation were prepared, with analytical measurements performed in triplicate for each batch. The optimized lyophilized liposomal batches were stored in a temperature-controlled chamber at 25 ± 2 °C and relative humidity of 60 ± 5% in tightly closed amber glass vials. Samples were analyzed immediately after lyophilization (0 months) and after 3, 6, and 12 months of storage. At each predetermined time point, the lyophilized formulations were reconstituted with purified water to their initial volume and characterized in terms of particle size, PDI, and ZP, as described in Section 3.7.3, and EE, morphological characterization by TEM and moisture content. All analyses were performed in triplicate.

The residual moisture content of the lyophilized liposomal formulations was determined by Karl Fischer titration using an SI Analytics TitroLine^®^ 7500 KF titrator. Approximately 15 mg of each lyophilized sample was accurately weighed and dissolved in a known volume of anhydrous methanol. An aliquot of the resulting solution was withdrawn using a gas-tight syringe and injected directly into the titration cell. The water content of the methanol blank was determined prior to sample analysis and subtracted from the measured values. All measurements were performed in triplicate, and the results were expressed as residual moisture content (% *w*/*w*).

### 3.11. Morphological Characterization of Lyophilized Liposomes by TEM

The morphology of the lyophilized liposomes after reconstitution was examined by transmission electron microscopy (TEM). Briefly, 1 mg of lyophilized liposomes was dispersed in 2 mL of Milli-Q^®^ water, and an aliquot of the resulting dispersion was deposited onto carbon-coated copper grids. Negative staining was performed using a 2% (*w*/*v*) aqueous phosphotungstic acid (PTA) solution, with a contact time of 1 min, followed by rinsing with water for 1 min. PTA was used to provide negative contrast for visualization of the vesicular structures, following the general principles described for TEM examination of lipid vesicles [91,92,93]. The samples were examined using a JEOL JEM-2010 transmission electron microscope operated at an accelerating voltage of 200 kV. TEM analyses were performed at the Electron Microscopy Service of the Universidade de Santiago de Compostela (USC), Santiago de Compostela, Spain.

### 3.12. In Vitro Cytocompatibility Assessment 

For cytotoxicity assessment, adult human dermal fibroblasts (Innoprot, Derio, Bizkaia, Spain; Cat. Nos. P10858) were used. The cells were rapidly thawed in a 37 °C water bath according to the manufacturer’s instructions. Adult human dermal fibroblasts were cultured in complete Dulbecco’s Modified Eagle Medium/Nutrient Mixture F-12 (DMEM/F-12; Gibco, Thermo Fisher Scientific, Waltham, MA, USA) supplemented with 10% fetal bovine serum (FBS; Gibco, Thermo Fisher Scientific, Waltham, MA, USA) and 1% penicillin–streptomycin (Gibco, Thermo Fisher Scientific, Waltham, MA, USA). Cells were maintained at 37 °C in a humidified incubator with 5% CO_2_ (ICO50med, Memmert GmbH + Co. KG, Schwabach, Germany). The cells were cultured in 75 cm^2^ vented tissue culture flasks (SORFA, Zhejiang, China) until they reached 70–80% confluence. The cultures were then washed with PBS without calcium and magnesium (VWR International GmbH, Vienna, Austria), and the cells were detached using 0.25% trypsin–EDTA solution (Innoprot, Derio, Bizkaia, Spain). The cells were then seeded into 96-well tissue culture plates at a density of 3 × 10^4^ cells/mL, with 100 μL of cell suspension per well. The following day, the samples of *C. cardunculus* var. *scolymus* extract and lyophilized liposomes loaded with *C. cardunculus* var. *scolymus* extract, previously sterilized by exposure to 70% ethanol for 45 min (50 mg of extract per 200 μL ethanol) and vortexed at 3000 rpm (Vortex Mixer V-1 Plus, BOECO, Germany) during 2 min, were suspended in complete cell culture medium. Following serial dilution, 100 μL of each extract solution was added to the respective wells to obtain final extract concentrations of 400, 300, 200, 100, 75, 50, 25, 10, and 5 μg/mL. All experimental conditions were performed in three independent experiments (n = 3). Cells cultured in complete medium without the tested extracts served as the control group (n = 8). The cytotoxicity of the tested samples was evaluated after 24, 48, and 72 h using the MTT assay (Sigma-Aldrich, Taufkirchen, Germany) [94,95]. At each time point, 20 μL of 0.5% MTT solution was added to each well, followed by incubation under standard culture conditions. After 3 h of incubation, the culture medium was carefully aspirated from each well using a vacuum aspirator (FTA-2i, Biosan, Latvia), taking care not to disturb the cell monolayer. Subsequently, 200 μL of isopropanol (Stan-Chem, Poland) was added to each well to dissolve the formazan crystals, and the plates were shaken on an orbital shaker (ES-20, Biosan, Riga, Latvia) at 120 rpm for 15 min. Following complete dissolution of the formazan crystals, the absorbance was measured at 570 nm using a spectrophotometer (Synergy H1 microplate reader (BioTek Instruments, Inc., Winooski, VT, USA), with isopropanol serving as the blank (n = 8).

### 3.13. Statistical Analysis

The results are presented as mean ± standard deviation (SD), as appropriate. Experimental design and model analysis were performed using MODDE 13 software (Sartorius Data Analytics AB, Umeå, Sweden). Partial Least Squares (PLS) regression was applied for model fitting, while analysis of variance (ANOVA) was used to assess model significance. The optimal formulation was identified using a desirability function approach. For the stability study, differences among storage time points were evaluated using one-way repeated-measures ANOVA, followed by Holm-adjusted pairwise comparisons. For the cytocompatibility assessment, relative cell viability was compared at each corresponding concentration and exposure time using Welch’s *t*-test. To account for multiple comparisons across concentrations at each exposure time, *p*-values were adjusted using the Holm method. Differences were considered statistically significant at adjusted *p* < 0.05.

## 4. Conclusions

This study demonstrates the applicability of an integrated QbD-guided strategy for the development of liposomal formulations containing *Cynara cardunculus* var. *scolymus* L. extract. By integrating the optimization of extract preparation with the systematic evaluation of formulation composition and processing conditions, the study established a coherent development framework linking the characteristics of the plant-derived payload to the critical quality attributes of the resulting liposomal system. Beyond the optimization of an individual formulation, the findings highlight the importance of considering extract preparation, formulation composition, processing conditions, and stabilization as interconnected elements governing the quality of liposomal systems containing complex plant extracts. Purification prior to payload assessment enabled characterization of the encapsulated fraction, while the release study provided further insight into the behavior of the phenolic payload under different release conditions. Importantly, lyophilization enabled preservation of the key physicochemical attributes upon reconstitution, with long-term stability assessment supporting the maintenance of the principal quality characteristics and vesicular morphology during storage. The preliminary cytocompatibility findings further supported the feasibility of the developed formulation while remaining exploratory in nature.

Collectively, these findings establish a formulation-development framework in which extraction, phytochemical characterization, liposomal composition, processing, purification, release behavior, and stabilization are considered as interconnected stages rather than isolated experimental steps. This integrated approach provides a scientifically grounded basis for the rational development of liposomal systems incorporating complex plant extracts, while the conclusions remain confined to the technological and preliminary biological evidence generated in the present study.

## Figures and Tables

**Figure 1:**
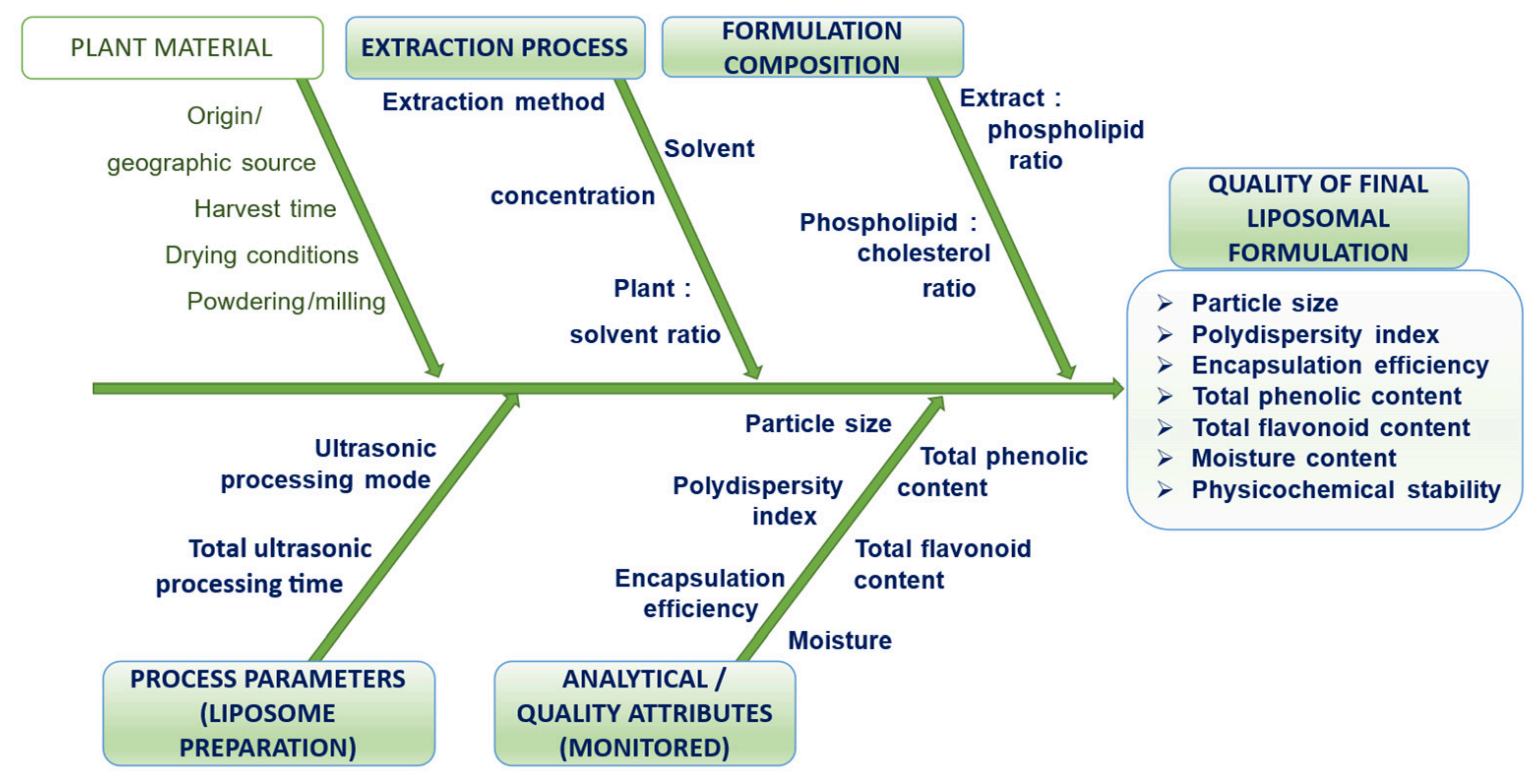
Ishikawa (fishbone) diagram identifying the potential Critical Material Attributes (CMAs) and Critical Process Parameters (CPPs) affecting the *Cynara cardunculus* var. *scolymus* L. extract-loaded liposomes. Variables experimentally investigated in the present study are highlighted in bold.

**Figure 2:**
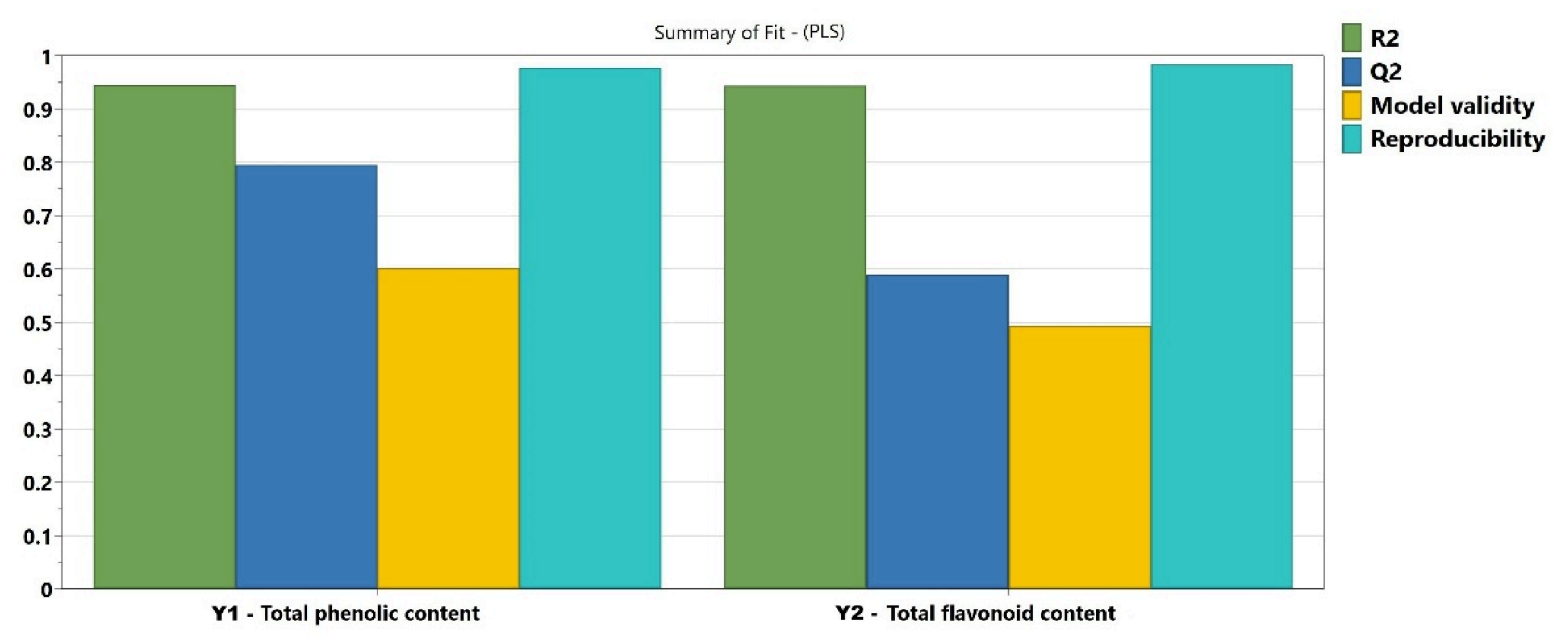
Model fitting and validation statistics for the developed partial least squares (PLS) models. Y1—total phenolic content (TPC); Y2—total flavonoid content (TFC).

**Figure 3:**
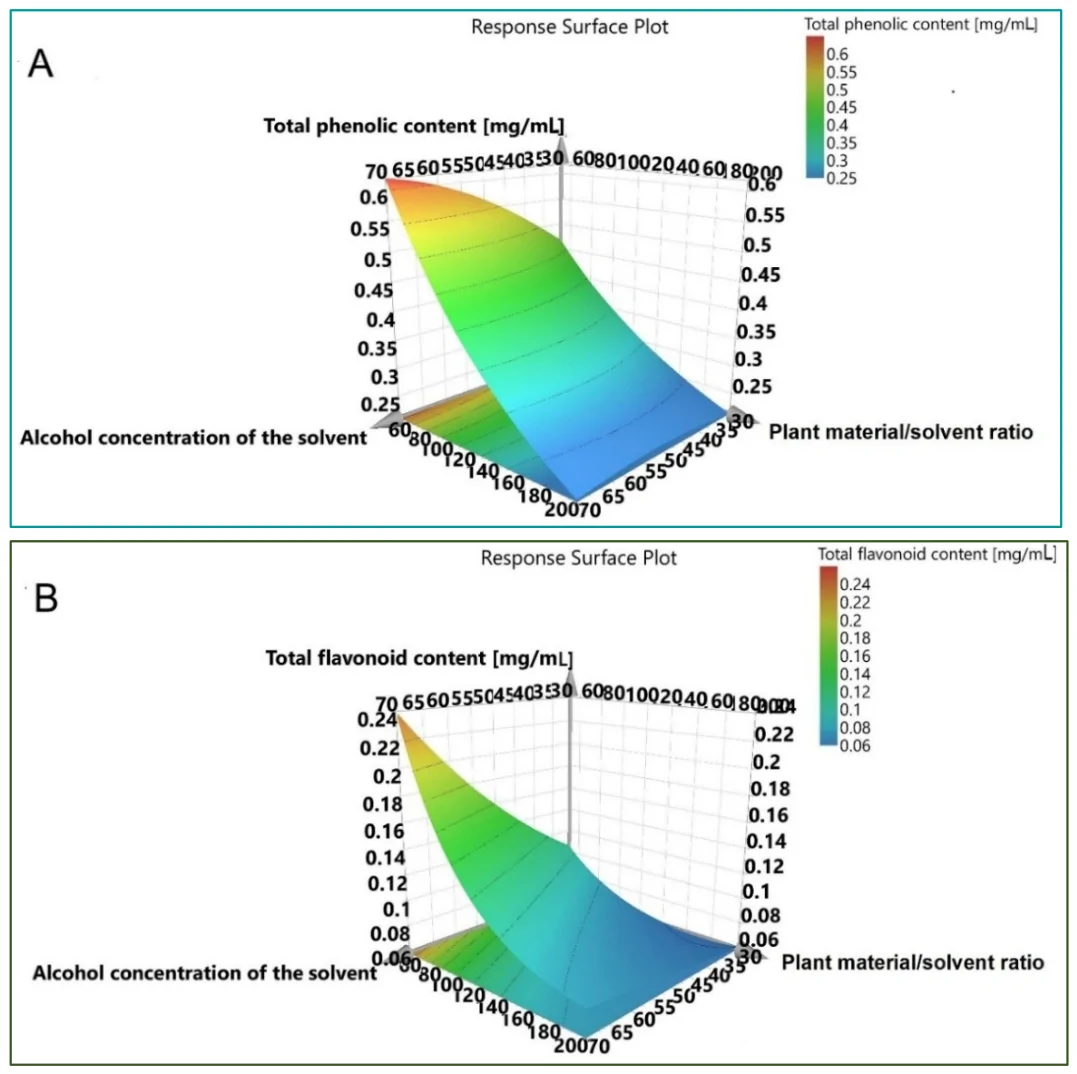
Response surface plots showing the combined effects of ethanol concentration and plant material-to-solvent ratio on the predicted (**A**) total phenolic content (TPC) and (**B**) total flavonoid content (TFC) under ultrasound-assisted extraction (UAE) conditions.

**Figure 4:**
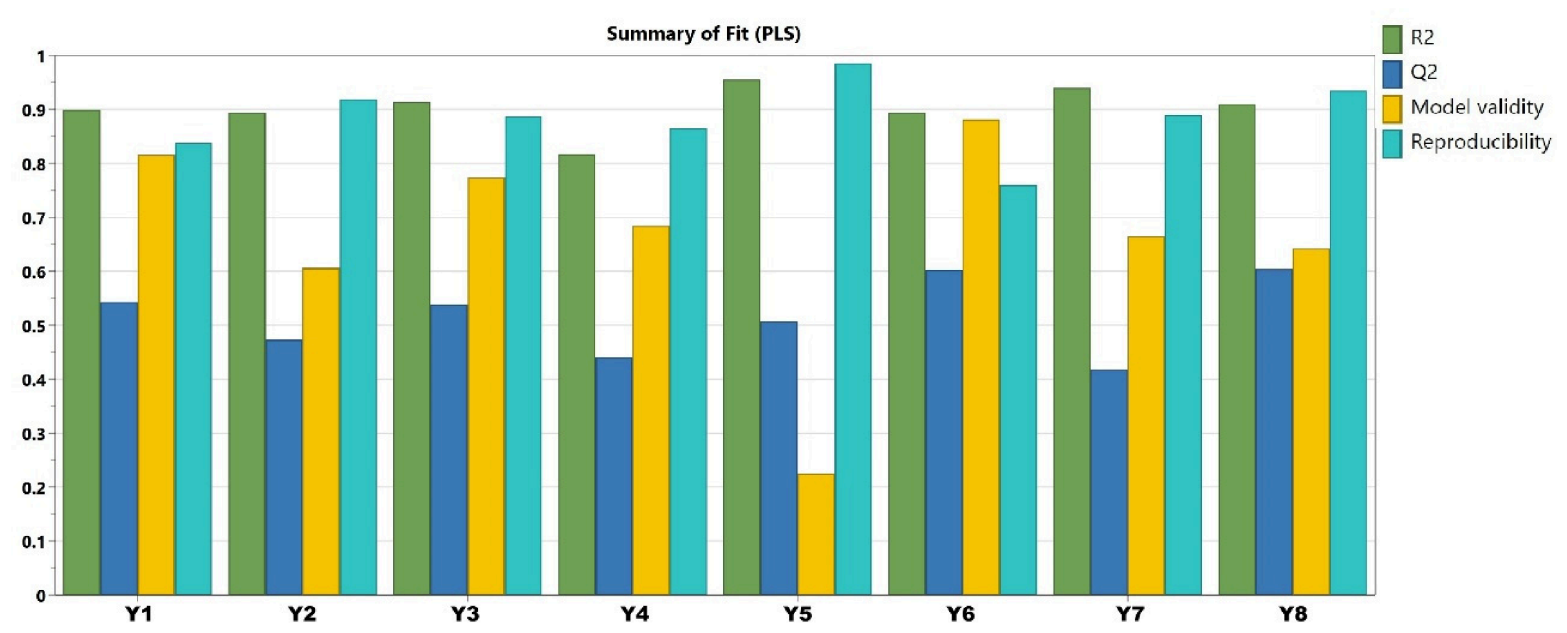
Summary of fit statistics for the partial least squares (PLS) models developed for the investigated responses. Abbreviations: Y1, particle size before sonication; Y2, particle size after sonication; Y3, PDI before sonication; Y4, PDI after sonication; Y5, EE before sonication; Y6, EE after sonication; Y7, DL before sonication; Y8, DL after sonication.

**Figure 5:**
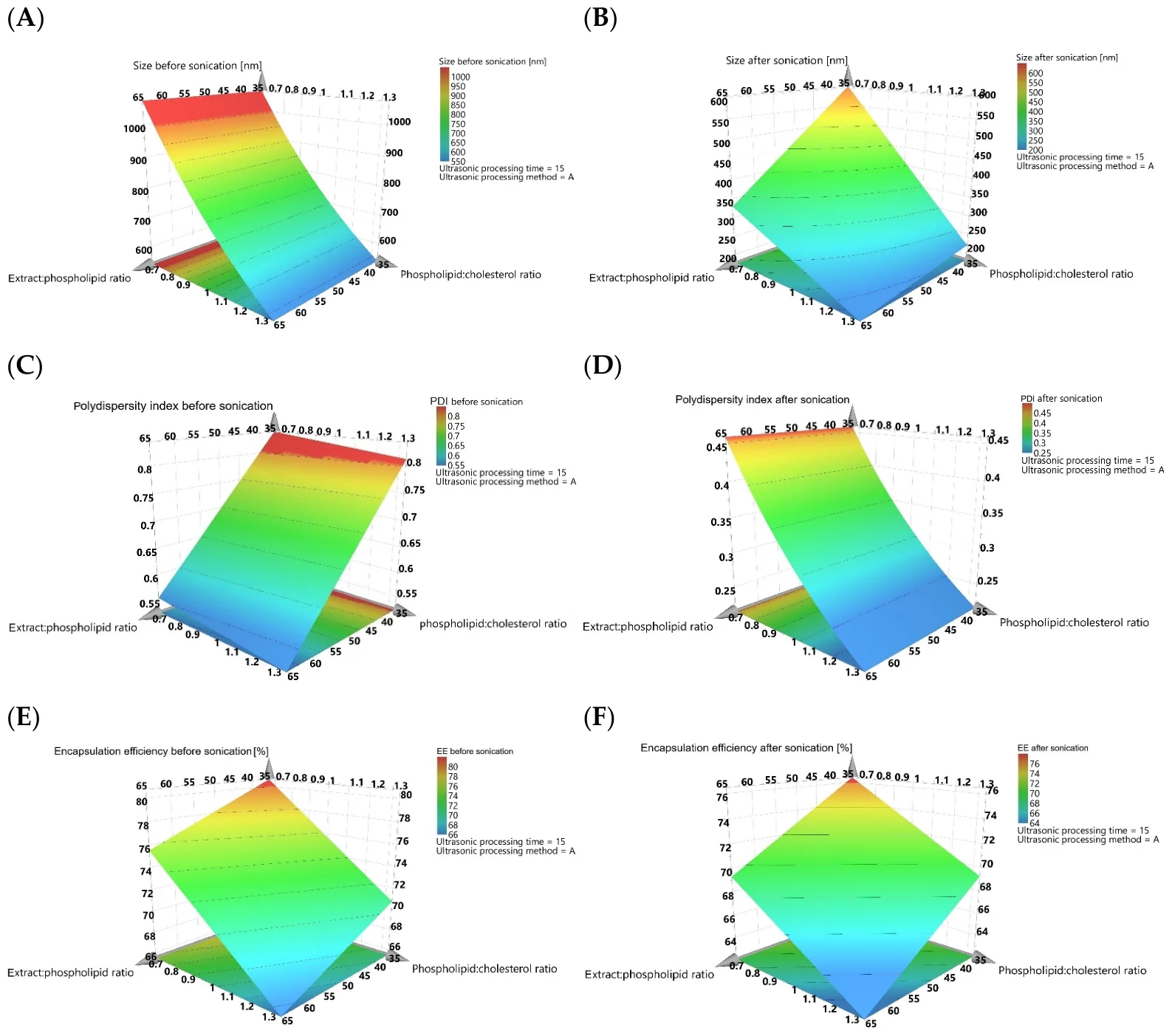

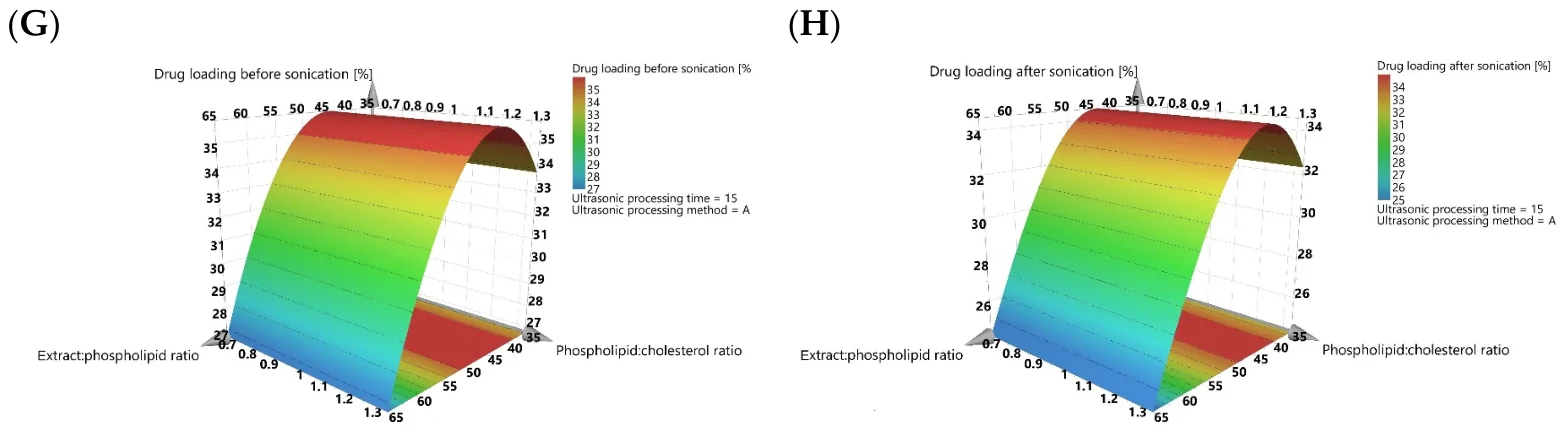
Response surface plots illustrating the combined effects of formulation composition and ultrasonic processing conditions on (**A**) particle size before sonication, (**B**) particle size after sonication, (**C**) polydispersity index (PDI) before sonication, (**D**) polydispersity index (PDI) after sonication, (**E**) encapsulation efficiency (EE) before sonication, (**F**) encapsulation efficiency (EE) after sonication, (**G**) drug loading before sonication, and (**H**) drug loading after sonication, with ultrasonic processing method fixed as Method A.

**Figure 6:**
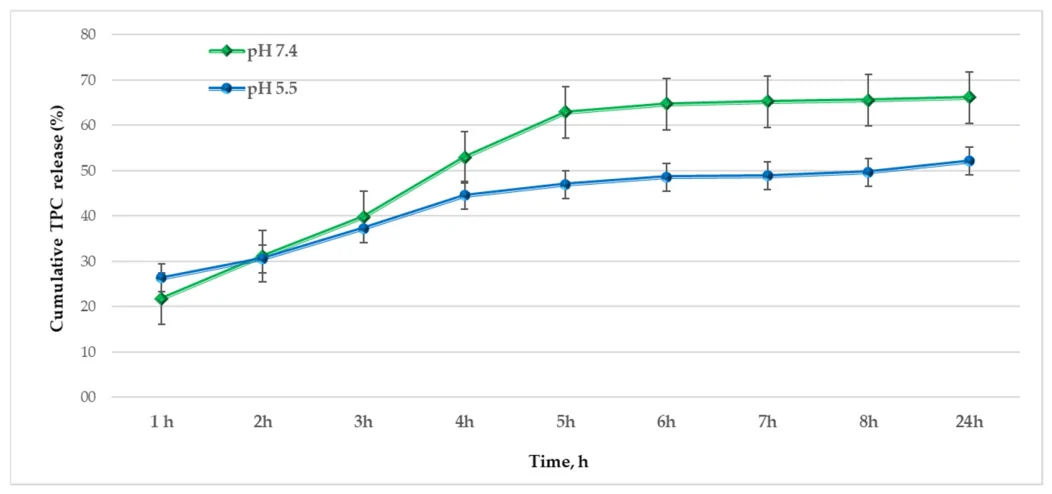
*In vitro* cumulative release profiles of phenolic compounds from the *Cynara cardunculus* var. *scolymus* L. extract-loaded liposomal formulation obtained under the optimized conditions at pH 5.5 and 7.4 over 24 h. Released phenolic compounds were quantified as total phenolic content (TPC) and expressed as gallic acid equivalents (GAEs). Data are presented as mean ± SD from three independent experiments (n = 3).

**Figure 7:**
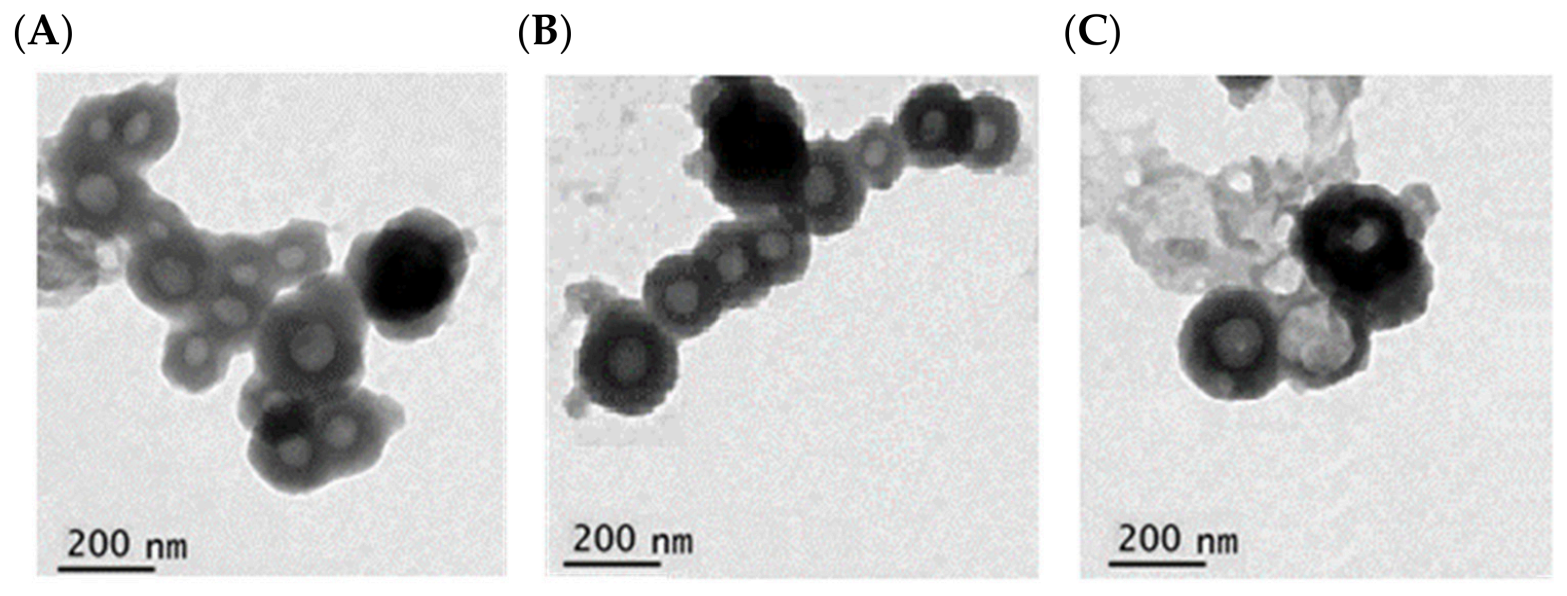
Transmission electron microscopy (TEM) images of lyophilized *C. cardunculus* var. *scolymus* extract-loaded liposomes after reconstitution following (**A**) 3, (**B**) 6, and (**C**) 12 months of storage. Scale bars: 200 nm.

**Figure 8:**
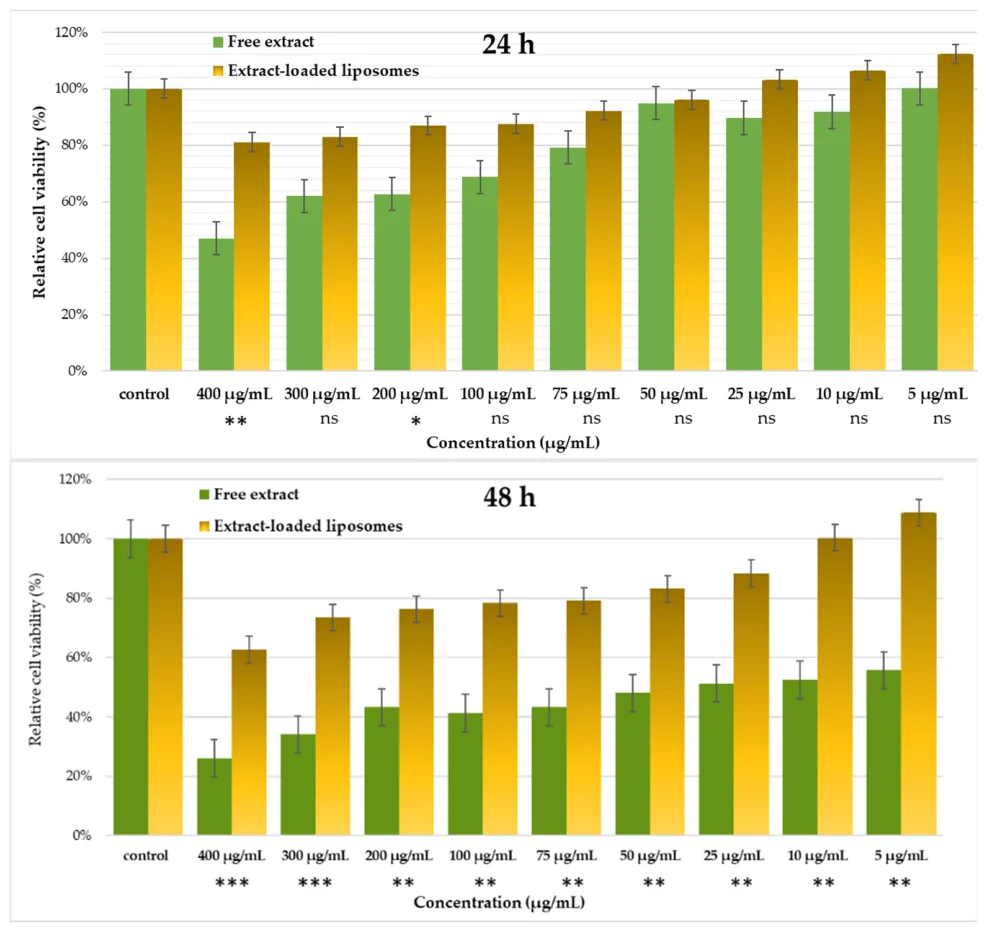

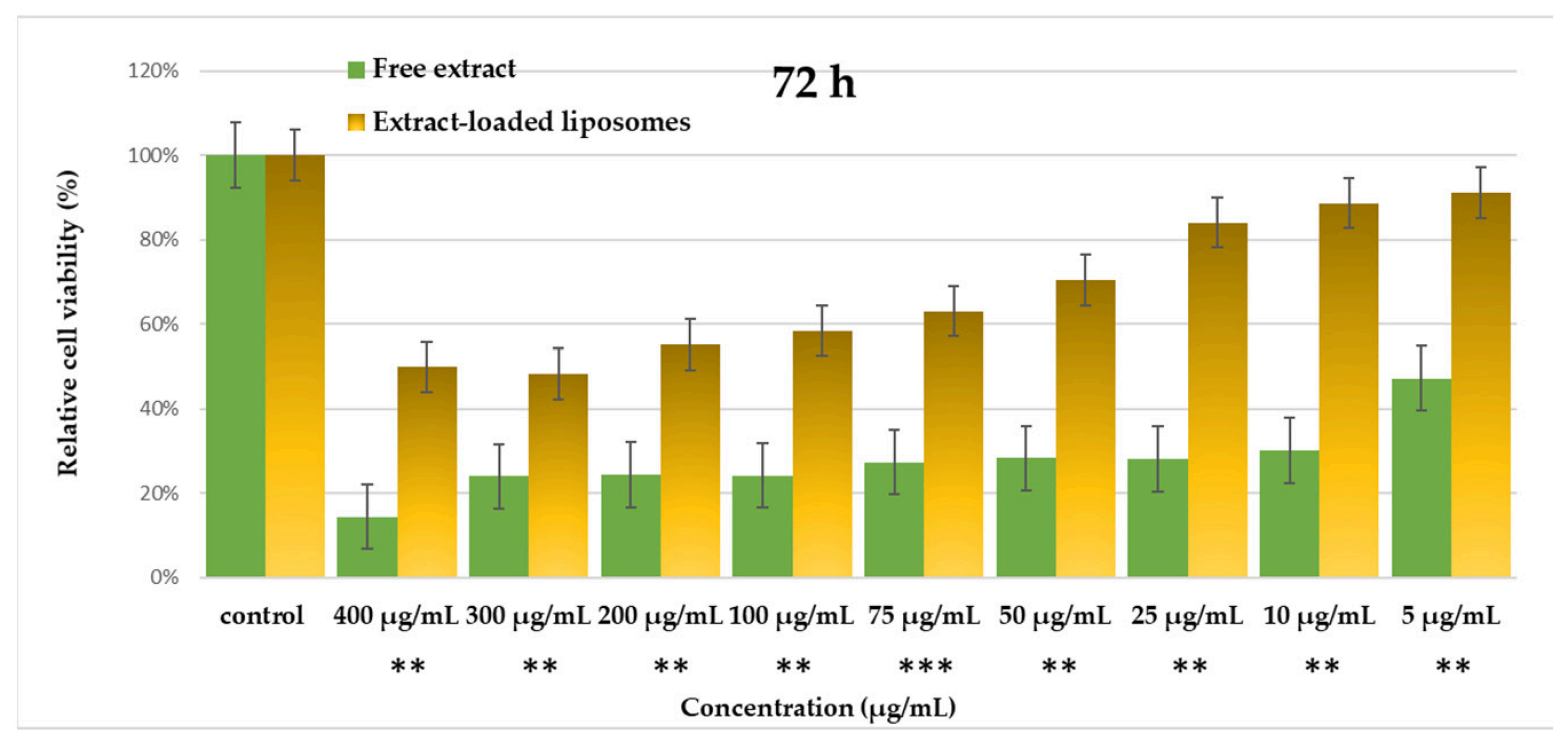
*In vitro* preliminary cytocompatibility of *Cynara cardunculus* var. *scolymus* L. free extract and extract-loaded liposomes toward adult human dermal fibroblasts after 24 h, 48 h, and 72 h of exposure, evaluated using the 3-(4,5-dimethylthiazol-2-yl)-2,5-diphenyltetrazolium bromide (MTT) assay. Data are presented as mean ± SD from three independent experiments (n = 3). Statistical comparisons between the free extract and extract-loaded liposomes at the corresponding concentration and exposure time were performed using Welch’s *t*-test, with *p*-values adjusted for multiple comparisons using the Holm method. ns, not significant (*p* ≥ 0.05); * *p* < 0.05; ** *p* < 0.01; *** *p* < 0.001.

**Table 1:** Phytochemical profile of the optimized *Cynara cardunculus* var. *scolymus* L. extract selected for subsequent liposomal encapsulation, determined by liquid chromatography–trapped ion mobility spectrometry–high-resolution tandem mass spectrometry (LC–TIMS-HRMS/MS).

No.	Putative Compound Identity	Class	RT (min)	[M-H]^−^ *m*/*z* exp.	Molecular Formula	CCS (Å^2^)
1	Quinic acid	Organic acid	0.88	191.05642	C_7_H_12_O_6_	139.4
2	Chlorogenic acid	Hydroxycinnamic acid	1.08	353.08814	C_16_H_18_O_9_	189.6
3	Quercetin 3,4′-dihexoside	Flavonol glycoside	1.13	625.14118	C_27_H_30_O_17_	233.2
4	*trans*-Caffeic acid	Hydroxycinnamic acid	1.2	179.03519	C_9_H_8_O_4_	136.3
5	Quercetin-*O*-hexoside	Flavonol glycoside	1.38	463.08841	C_21_H2_0_O_12_	204.2
6	Kaempferol-*O*-rutinoside (Nicotiflorin)	Flavonol glycoside	1.39	593.15144	C_27_H_30_O_15_	230.5
7	*p*-coumaric acid	Hydroxycinnamic acid	1.53	163.04032	C_9_H_8_O_3_	133.5
8	Vanillic acid	Phenolic acid	1.74	167.03519	C_8_H_8_O_4_	133.2
9	Kaempferol-*O*-hexoside	Flavonol glycoside	1.83	447.09316	C_21_H_20_O_11_	203.4
10	Dicaffeoylquinic acid isomer (Isochlorogenic acid A)	Dicaffeoylquinic acid	1.86	515.11937	C_25_H_24_O_12_	213.2
11	Quercetin-*O*-deoxyhexoside	Flavonol glycoside	2.16	447.09361	C_21_H_20_O_11_	203.9
12	Hydroxy-methoxycinnamic acid (Isoferulic acid)	Phenolic acid	3.01	193.05081	C_10_H_10_O_4_	143.5
13	Kaempferol-*O*-deoxyhexoside	Flavonol glycoside	3.44	431.09912	C_21_H_20_O_10_	202.6
14	Quercetin	Flavonol	5.24	301.03617	C_15_H_10_O_7_	167.6
15	Kaempferol	Flavonol	10.41	285.04085	C_15_H_10_O_6_	165.2

Note: RT, retention time; m/z, mass-to-charge ratio; CCS, collision cross section.

**Table 2:** Critical quality attributes (CQAs) of lyophilized *Cynara cardunculus* var. *scolymus* L. extract-loaded liposomes during storage at 25 °C.

Storage Time	Size (nm)	Polydispersity Index	Zeta Potential (mV)	Encapsulation Efficiency, (%)	Moisture, (%)
Prior to lyophilization	224.1± 1.9	0.206 ± 0.014	−2.205 ±0.01	75.95 ± 0.24	n.a.
0 months	224.3 ± 4.0 ^a^	0.203 ± 0.021 ^a^	−2.206 ± 0.16 ^a^	75.92 ± 1.09 ^a^	0.80 ± 0.11 ^a^
3 months	241.0 ± 3.6 ^a^	0.221 ± 0.003 ^a^	−1.924 ± 0.02 ^ab^	75.80 ± 1.16 ^a^	0.69 ± 0.52 ^a^
6 months	242.3 ± 2.5 ^a^	0.223 ± 0.011 ^a^	−1.941 ± 0.08 ^ab^	75.76 ± 0.73 ^a^	1.11 ± 0.06 ^a^
12 months	290.3 ± 4.2 ^b^	0.252 ± 0.007 ^a^	−1.851 ± 0.11 ^b^	73.25 ± 2.11 ^a^	1.45 ± 0.24 ^a^

Note: Values are expressed as mean ± SD (n = 3 independent batches). “Prior to lyophilization” refers to the purified, freshly prepared optimized liposomal formulation, whereas 0 months refers to the formulation analyzed immediately after lyophilization and reconstitution. Statistical comparisons among storage time points (0, 3, 6, and 12 months) were performed using one-way repeated-measures ANOVA followed by Holm-adjusted pairwise comparisons. Different superscript letters within the same column indicate significant pairwise differences (adjusted *p* < 0.05). Pre-lyophilization values were not included in the storage-time analysis. n.a., not applicable.

**Table 3:** D-optimal experimental design matrix for optimization of *Cynara cardunculus* var. *scolymus* L. extraction.

Exp Name	Run Order	X_1_	X_2_	X_3_
N1	3	Ultrasonication	1:50	30
N2	2	Ultrasonication	1:50	70
N3	11	Turbo Extraction	1:50	30
N4	14	Turbo Extraction	1:50	70
N5	18	Turbo Extraction	1:50	50
N6	17	Maceration	1:50	30
N7	13	Maceration	1:50	70
N8	21	Ultrasonication	1:100	50
N9	16	Turbo Extraction	1:100	30
N10	7	Turbo Extraction	1:100	70
N11	19	Maceration	1:100	50
N12	5	Ultrasonication	1:200	30
N13	20	Ultrasonication	1:200	70
N14	9	Turbo Extraction	1:200	30
N15	1	Turbo Extraction	1:200	70
N16	6	Turbo Extraction	1:200	50
N17	15	Maceration	1:200	30
N18	12	Maceration	1:200	70
N19	8	Maceration	1:200	50
N20	4	Maceration	1:200	50
N21	10	Maceration	1:200	50

Note: X_1_, extraction method; X_2_, plant-to-solvent ratio (g/mL); X_3_, ethanol concentration (%).

**Table 4:** Experimental design matrix for liposomes loaded with *Cynara cardunculus* var. *scolymus* L. extract.

Exp No	Exp Name	Run Order	Incl/Excl	X1	X2	X3	X4
1	N1	2	Incl	65	1:2	A	9
2	N2	7	Incl	35	2:1	A	9
3	N3	12	Incl	35	1:2	A	21
4	N4	11	Incl	65	2:1	A	21
5	N5	8	Incl	35	1:2	B	9
6	N6	15	Incl	65	2:1	B	9
7	N7	5	Incl	65	1:2	B	21
8	N8	13	Incl	35	2:1	B	21
9	N9	1	Incl	65	1:2	C	9
10	N10	10	Incl	35	2:1	C	9
11	N11	3	Incl	35	1:2	C	21
12	N12	4	Incl	65	2:1	C	21
13	N13	9	Incl	50	1:1	C	15
14	N14	14	Incl	50	1:1	C	15
15	N15	6	Incl	50	1:1	C	15

Note: X1—plant extract amount (mg), with the corresponding extract:phospholipid compositions of 35:65, 50:50, and 65:35 (*w*/*w*); X2—phospholipid-to-cholesterol ratio, with experimental levels of 1:2, 1:1, and 2:1; X3—ultrasonic processing method (A—probe sonication; B—combined sonication and ultrasonic bath treatment; C—ultrasonic bath treatment); X4—total ultrasonic processing time (min).

## Data Availability

Data are contained within the article.

## Supplementary Materials

The following supporting information can be downloaded at https://www.mdpi.com/article/10.3390/molecules31193407/s1. Figure S1: Standardized coefficient plot showing the main and interaction effects of the investigated extraction variables on total phenolic content (TPC). Error bars represent the confidence intervals of the estimated model coefficients. X1, extraction method; X2, plant material-to-solvent ratio; X3, ethanol concentration; TPC, total phenolic content (mg/mL); Figure S2: Standardized coefficient plot showing the main and interaction effects of the investigated extraction variables on total flavonoid content (TFC). Error bars represent the confidence intervals of the estimated model coefficients. X1, extraction method; X2, plant material-to-solvent ratio; X3, ethanol concentration; TFC, total flavonoid content (mg/mL); Figure S3: Standardized coefficient plots showing the effects of the investigated formulation and process variables and their interactions on particle size (A) before and (B) after sonication. Error bars represent 95% confidence intervals. X1, extract-to-phospholipid ratio; X2, phospholipid-to-cholesterol ratio; X3, ultrasonic processing method (A, probe sonication; B, combined probe sonication and ultrasonic bath treatment; C, ultrasonic bath treatment); X4, total ultrasonic processing time (min); Figure S4: Standardized coefficient plots showing the effects of the investigated formulation and process variables and their interactions on polydispersity index (PDI) (A) before and (B) after sonication. Error bars represent 95% confidence intervals. X1, extract-to-phospholipid ratio; X2, phospholipid-to-cholesterol ratio; X3, ultrasonic processing method (A, probe sonication; B, combined probe sonication and ultrasonic bath treatment; C, ultrasonic bath treatment); X4, total ultrasonic processing time (min); Figure S5: Standardized coefficient plots showing the effects of the investigated formulation and process variables and their interactions on drug loading (A) before and (B) after sonication. Error bars represent 95% confidence intervals. X1, extract-to-phospholipid ratio; X2, phospholipid-to-cholesterol ratio; X3, ultrasonic processing method (A, probe sonication; B, combined probe sonication and ultrasonic bath treatment; C, ultrasonic bath treatment); X4, total ultrasonic processing time (min); Figure S6: Standardized coefficient plots showing the effects of the investigated formulation and process variables and their interactions on encapsulation efficiency (EE) (A) before and (B) after sonication. Error bars represent 95% confidence intervals. X1, extract-to-phospholipid ratio; X2, phospholipid-to-cholesterol ratio; X3, ultrasonic processing method (A, probe sonication; B, combined probe sonication and ultrasonic bath treatment; C, ultrasonic bath treatment); X4, total ultrasonic processing time (min).
