# Supplementary material for: Quality by Design-Guided Development of Cynara cardunculus var. scolymus L. Extract-Loaded Liposomes
Source: Molecules. 2026 Sep 24;31(19):3407. doi: 10.3390/molecules31193407 (PMC13666192; doi:10.3390/molecules31193407)
Supplement: Supplementary file 1 [file molecules-31-03407-s001.zip › molecules-4468117-supplementary.pdf]

Supplementary Results from the Experimental Design and Optimization Studies

Section S1. Extraction Process Optimization

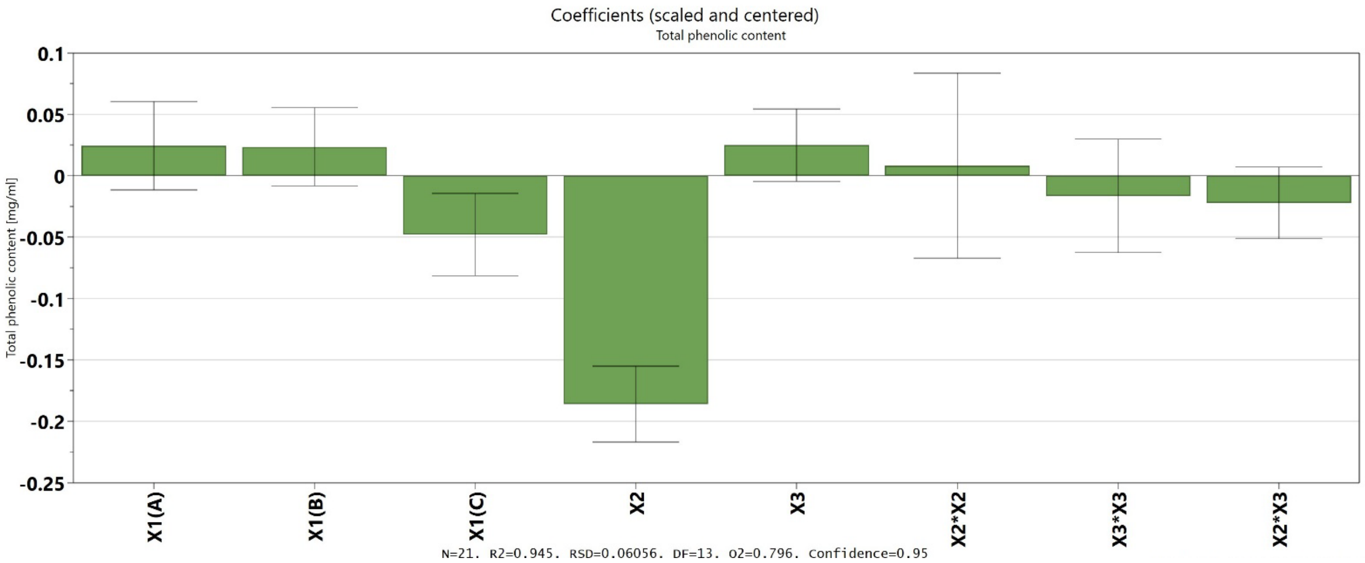

Figure S1. Standardized coefficient plot showing the main and interaction effects of the investigated extraction variables on total phenolic content (TPC). Error bars represent the confidence intervals of the estimated model coefficients. X1, extraction method; X2, plant material-to-solvent ratio; X3, ethanol concentration; TPC, total phenolic content (mg/mL).

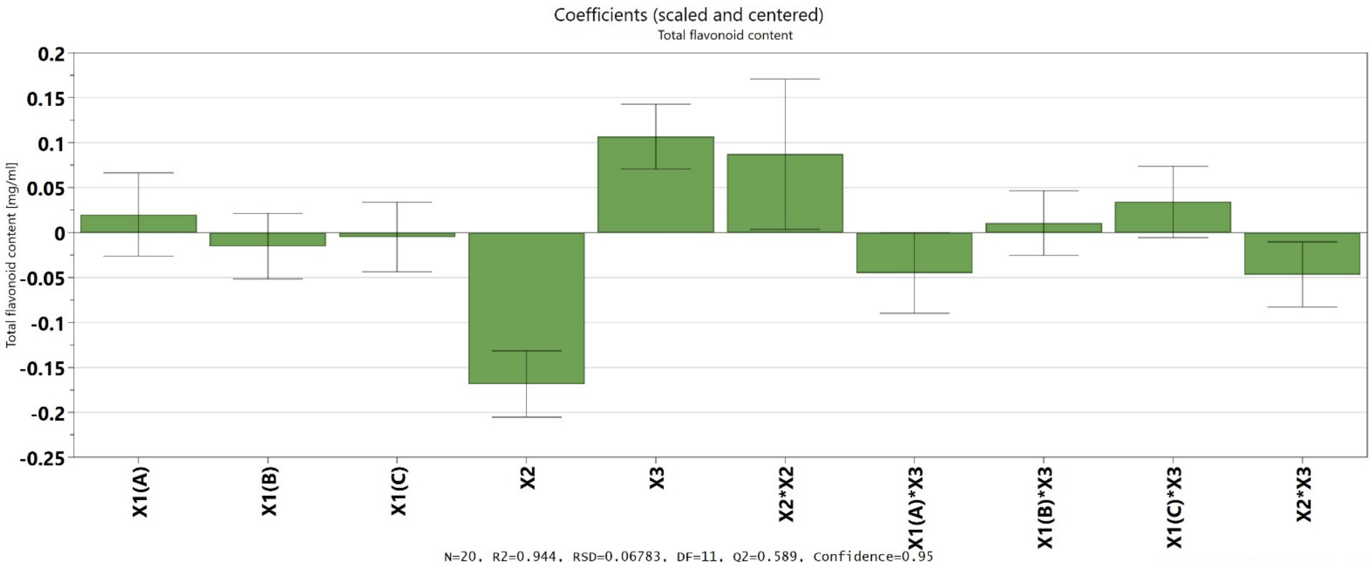

Figure S2. Standardized coefficient plot showing the main and interaction effects of the investigated extraction variables on total flavonoid content (TFC). Error bars represent the confidence intervals of the estimated model coefficients. X1, extraction method; X2, plant material-to-solvent ratio; X3, ethanol concentration; TFC, total flavonoid content (mg/mL).

Section S2. Liposomal Formulation and Process Optimization

A

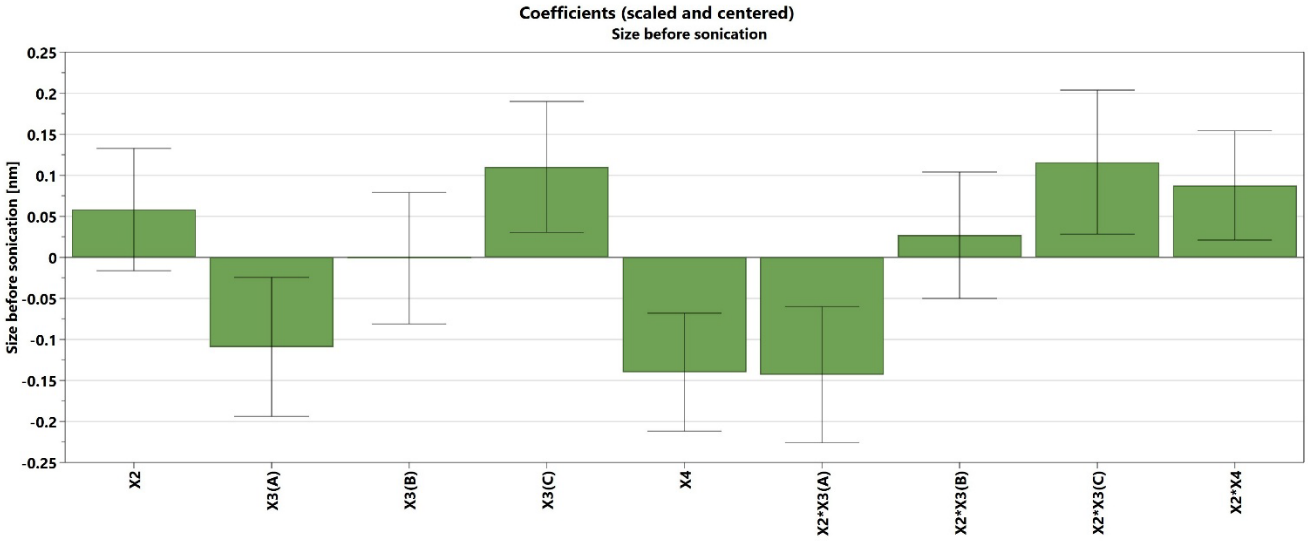

B

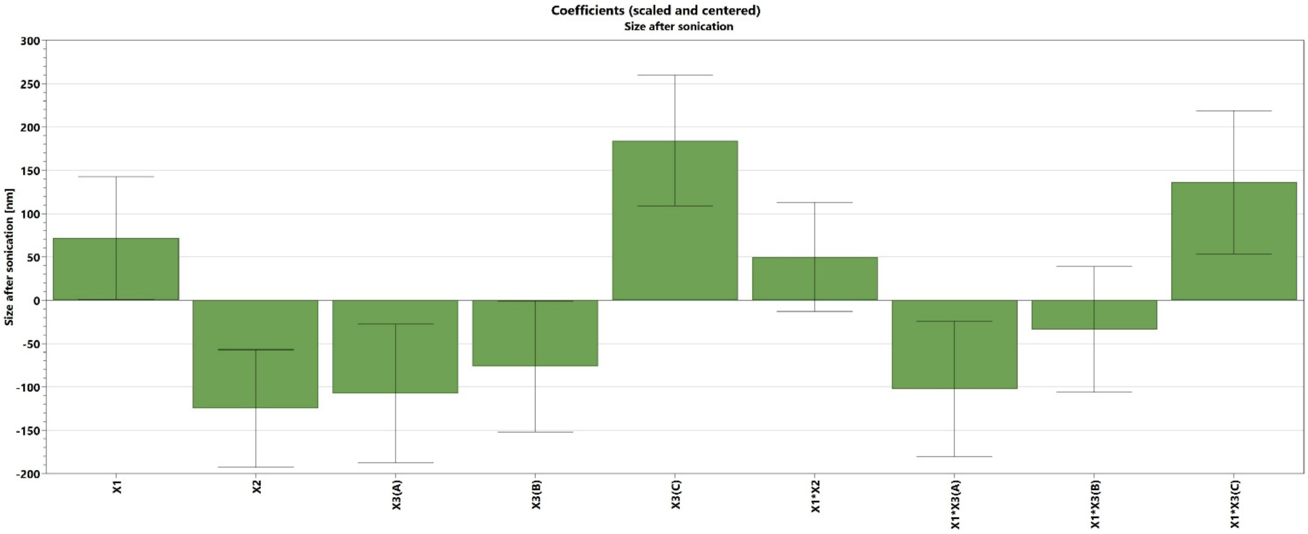

Figure S3. Standardized coefficient plots showing the effects of the investigated formulation and process variables and their interactions on particle size (A) before and (B) after sonication. Error bars represent 95% confidence intervals. X1, extract-to-phospholipid ratio; X2, phospholipid-to-cholesterol ratio; X3, ultrasonic processing method (A, probe sonication; B, combined probe sonication and ultrasonic bath treatment; C, ultrasonic bath treatment); X4, total ultrasonic processing time (min).

A

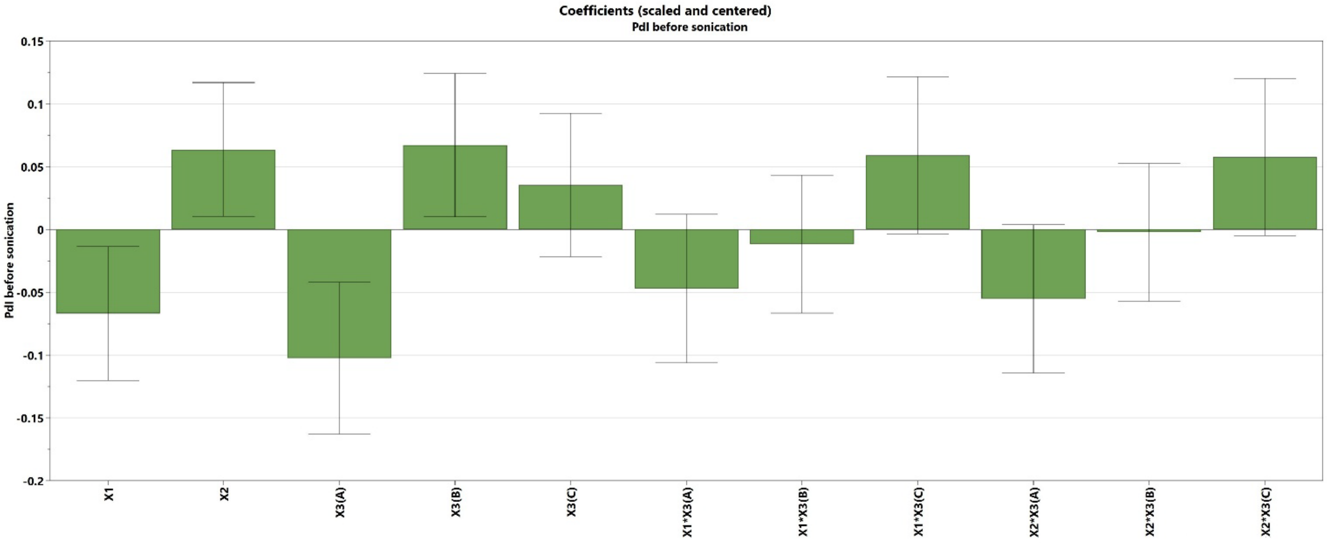

B

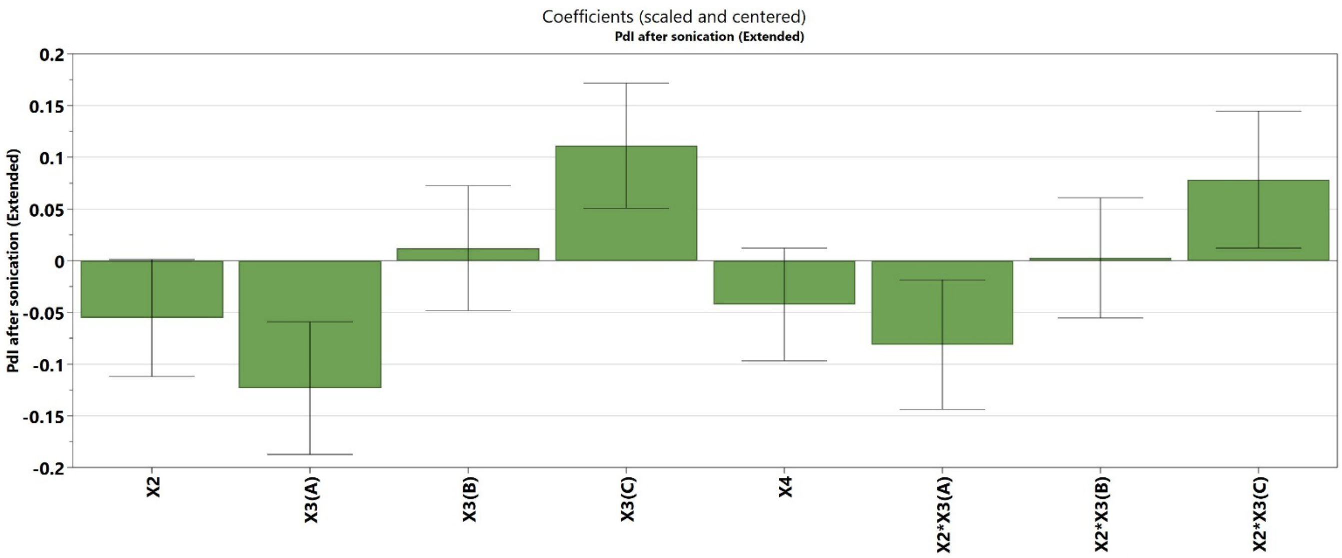

Figure S4. Standardized coefficient plots showing the effects of the investigated formulation and process variables and their interactions on polydispersity index (PDI) (A) before and (B) after sonication. Error bars represent 95% confidence intervals. X1, extract-to-phospholipid ratio; X2, phospholipid-to-cholesterol ratio; X3, ultrasonic processing method (A, probe sonication; B, combined probe sonication and ultrasonic bath treatment; C, ultrasonic bath treatment); X4, total ultrasonic processing time (min).

A

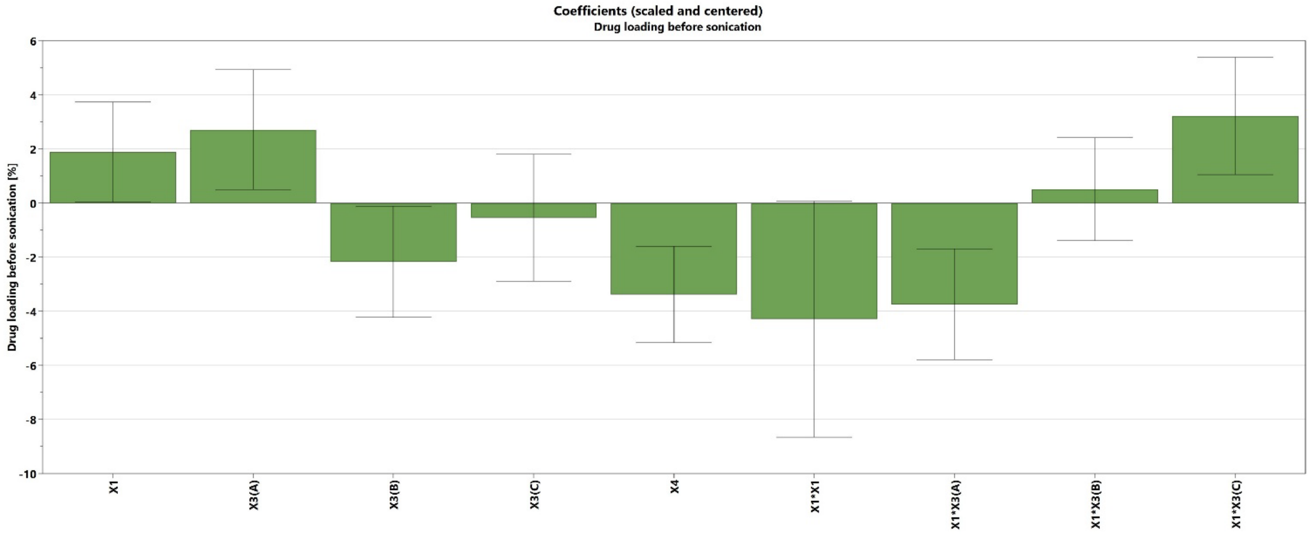

B

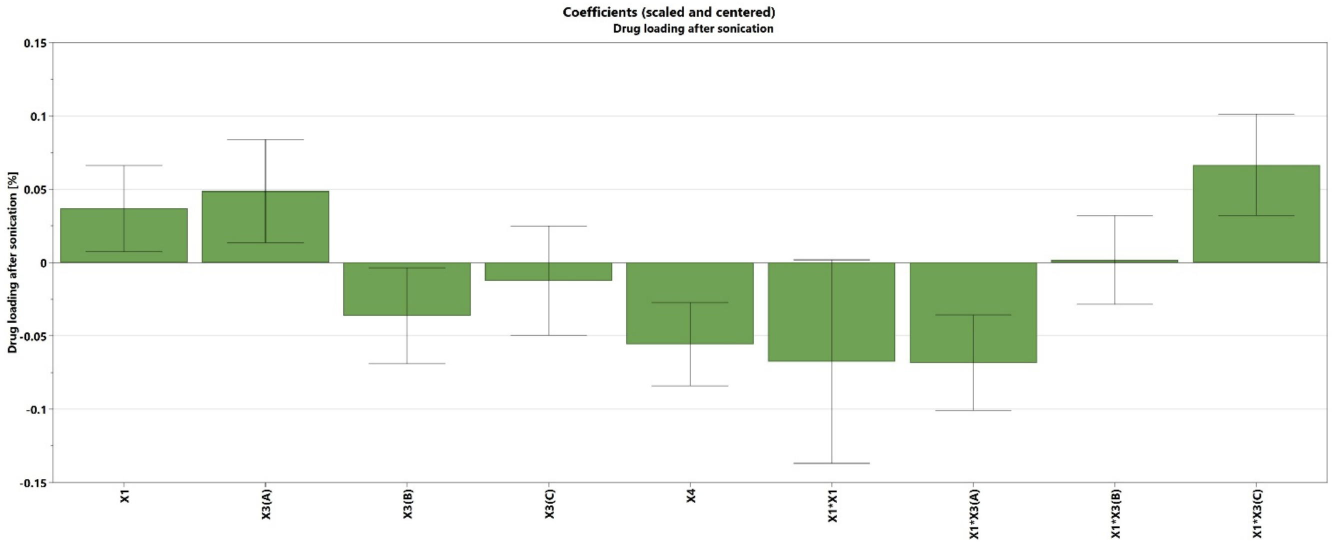

Figure S5. Standardized coefficient plots showing the effects of the investigated formulation and process variables and their interactions on drug loading (A) before and (B) after sonication. Error bars represent 95% confidence intervals. X1, extract-to-phospholipid ratio; X2, phospholipid-to-cholesterol ratio; X3, ultrasonic processing method (A, probe sonication; B, combined probe sonication and ultrasonic bath treatment; C, ultrasonic bath treatment); X4, total ultrasonic processing time (min).

A

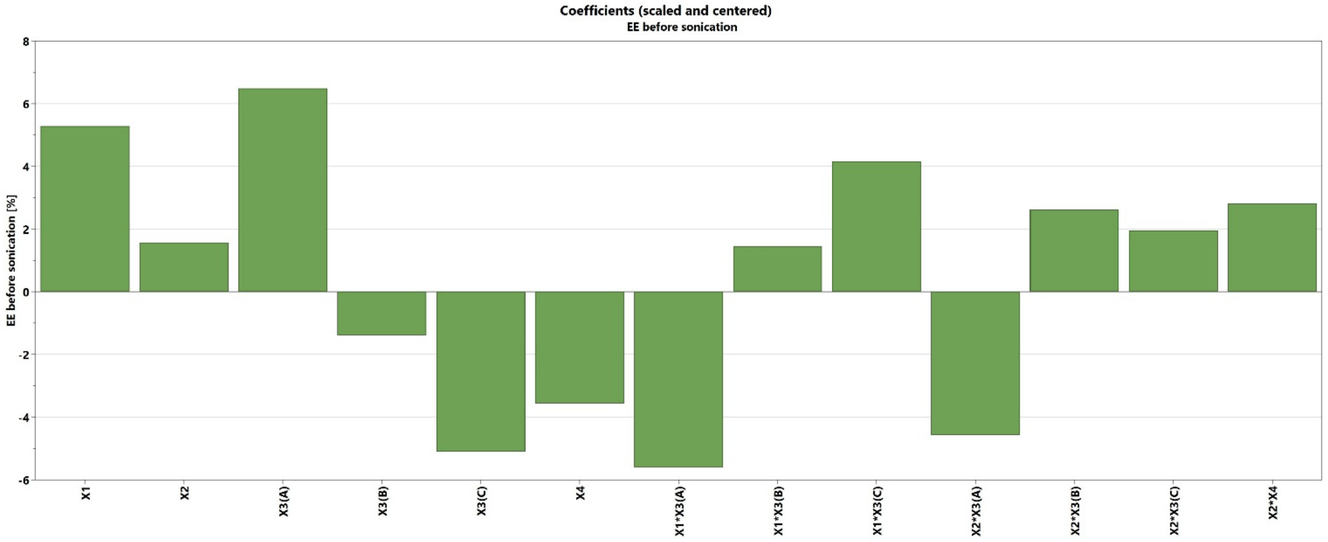

B

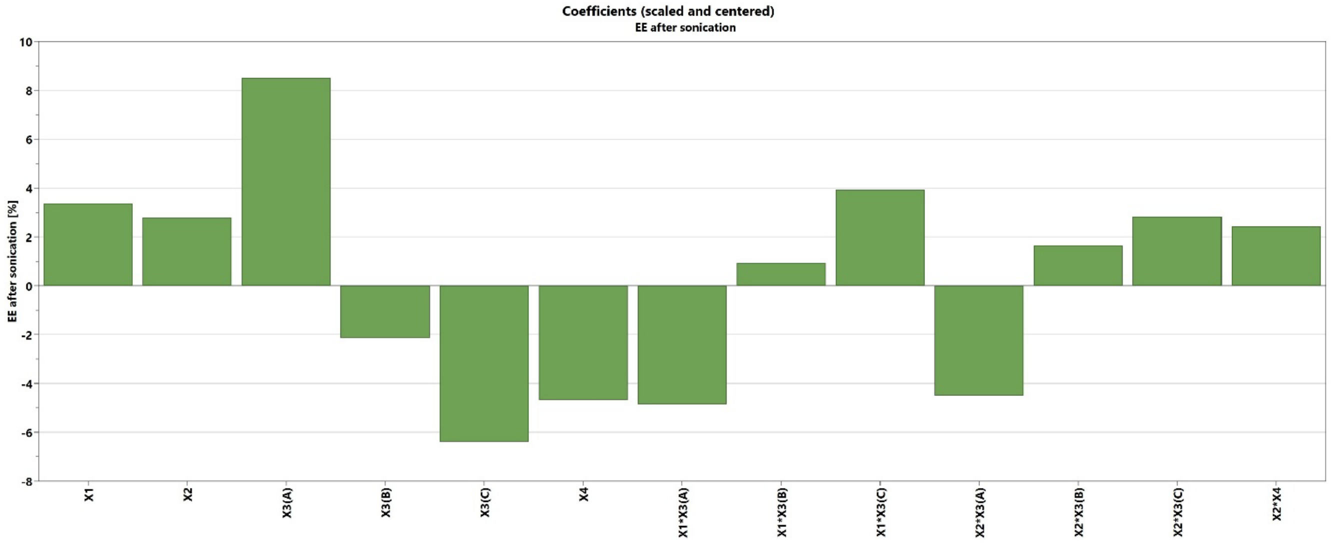

Figure S6. Standardized coefficient plots showing the effects of the investigated formulation and process variables and their interactions on encapsulation efficiency (EE) (A) before and (B) after sonication. Error bars represent 95% confidence intervals. X1, extract-to-phospholipid ratio; X2, phospholipid-to-cholesterol ratio; X3, ultrasonic processing method (A, probe sonication; B, combined probe sonication and ultrasonic bath treatment; C, ultrasonic bath treatment); X4, total ultrasonic processing time (min).
